# Narrow and Broad γ Bands Process Complementary Visual Information in Mouse Primary Visual Cortex

**DOI:** 10.1523/ENEURO.0106-21.2021

**Published:** 2021-11-02

**Authors:** Nicolò Meneghetti, Chiara Cerri, Elena Tantillo, Eleonora Vannini, Matteo Caleo, Alberto Mazzoni

**Affiliations:** 1 The Biorobotics Institute, Scuola Superiore Sant’Anna, Pisa 56025, Italy; 2 Department of Excellence for Robotics and AI, Scuola Superiore Sant’Anna, Pisa 56025, Italy; 3 Neuroscience Institute, National Research Council (CNR), Pisa 56124, Italy; 4 Fondazione Umberto Veronesi, Milan 20122, Italy; 5 Department of Pharmacy, University of Pisa, Pisa 56126, Italy; 6 Fondazione Pisana per la Scienza Onlus (FPS), Pisa 56017, Italy; 7 Scuola Normale Superiore, Pisa 56100, Italy; 8 Department of Biomedical Sciences, University of Padua, Padua 35131, Italy

**Keywords:** broad γ band, local field potential, narrow γ band, spiking neuronal network, visual contrast, visual cortex

## Abstract

γ Band plays a key role in the encoding of visual features in the primary visual cortex (V1). In rodents V1 two ranges within the γ band are sensitive to contrast: a broad γ band (BB) increasing with contrast, and a narrow γ band (NB), peaking at ∼60 Hz, decreasing with contrast. The functional roles of the two bands and the neural circuits originating them are not completely clear yet. Here, we show, combining experimental and simulated data, that in mice V1 (1) BB carries information about high contrast and NB about low contrast; (2) BB modulation depends on excitatory-inhibitory interplay in the cortex, while NB modulation is because of entrainment to the thalamic drive. In awake mice presented with alternating gratings, NB power progressively decreased from low to intermediate levels of contrast where it reached a plateau. Conversely, BB power was constant across low levels of contrast, but it progressively increased from intermediate to high levels of contrast. Furthermore, BB response was stronger immediately after contrast reversal, while the opposite held for NB. These complementary modulations were reproduced by a recurrent excitatory-inhibitory leaky integrate-and-fire network provided that the thalamic inputs were composed of a sustained and a periodic component having complementary sensitivity ranges. These results show that in rodents the thalamic-driven NB plays a specific key role in encoding visual contrast. Moreover, we propose a simple and effective network model of response to visual stimuli in rodents that might help in investigating network dysfunctions of pathologic visual information processing.

## Visual Abstract

**Figure:**
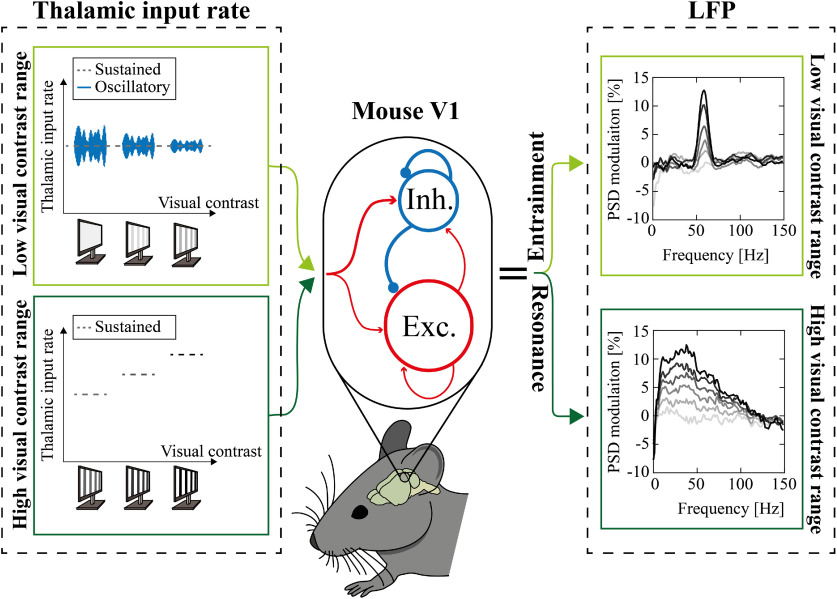

## Significance Statement

γ Oscillations are known to play a relevant and functional role in visual information processing. In the visual cortex of the mice two different frequency bands within this range have been found to display different sensitivity to visual stimuli. Here, we help understanding this peculiar phenomenon with two advancements. First, we characterize the response to visual contrast of the two bands, finding them to be complementary both in their temporal activation and in their sensitivity to contrasts. Second, we developed a spiking neurons network model showing that two complementary neural mechanisms originate the two bands. This suggests that these γ oscillations can be considered as two separate, yet complementary, information channels processing different aspects of the external world.

## Introduction

Neuronal γ band ([30–100] Hz) synchronization is a widespread functional mode in the mammalian cortex, known to improve long-range information transmission (König et al., 1995; Sohal, 2016). Coherently, γ synchronization contributes to the processing of different modality of sensory information, from audition (Brosch et al., 2002; Tsunada and Eliades, 2020) to olfaction (Beshel et al., 2007; Lepousez and Lledo, 2013), touch (Siegle et al., 2014), and nociception (Tan et al., 2019; Heid et al., 2020).

The role of γ synchronization in the processing of visual stimuli has been investigated for decades (Gray and Singer, 1989; Gray et al., 1989). Indeed, visual stimulus features are well known for modulating the power and the central frequency of neocortical oscillations in the γ range. Previous studies adopting different animal models (e.g., mice, cats, human and nonhuman primates) have indeed highlighted γ power to be critically dependent on orientation (Gray and Singer, 1989; Berens et al., 2008; Onorato et al., 2020), size (Gieselmann and Thiele, 2008; Perry et al., 2013), speed (Gray et al., 1989; Friedman-Hill et al., 2000; Womelsdorf et al., 2006), direction (Liu and Newsome, 2006), and contrast (Logothetis et al., 2001; Henrie and Shapley, 2005; Ray and Maunsell, 2010; Saleem et al., 2017; McAfee et al., 2018; Bartoli et al., 2019) of the visual stimulus.

Such cortical γ band oscillations originate from the coordinated interaction of excitation and inhibition (Sohal et al., 2009; Cardin, 2016) and might be detected by local field potential (LFP), although single unit activity is irregular (Buzsáki and Wang, 2012). Many modeling works have indeed described how γ synchronization originates in excitatory-inhibitory networks and how it is affected by external stimuli (Wilson and Cowan, 1972; Leung, 1982; Ermentrout and Kopell, 1998; Brunel and Wang, 2003; Börgers and Kopell, 2003; Geisler et al., 2005). Specifically, several studies focused on how such networks could capture contrast-induced modulation of γ band in primary visual cortex (V1; Mazzoni et al., 2008, 2010, 2011; Battaglia and Hansel, 2011; Jia et al., 2013; Roberts et al., 2013; Barbieri et al., 2014; Lowet et al., 2015; Zachariou et al., 2021). These works captured a variety of properties of γ band in the visual cortex of primates, but recent evidence showed that properties and the functional role of γ band in the visual cortex of rodents might display some peculiar differences. In particular, a very narrow γ band (NB) close to 60 Hz has been observed in the lateral geniculate nucleus (LGN) firing activity of awake mice (Saleem et al., 2017; Storchi et al., 2017) but has not been observed in primates. In a recent work (Saleem et al., 2017) this has been shown to induce a cortical narrow band oscillation at ∼60 Hz co-existing with the broad band activity in the 30- to 90-Hz range but with a different functional role. While the cortical broad band power increased with contrast, as in primates, the cortical narrow band power increased with luminance and decreased with contrast (Saleem et al., 2017). This suggests that in mice cortical γ band might have two components with specific frequency ranges and different encoding properties, marking a relevant difference from the γ band in the visual cortex of primates (see also Discussion, NB and BB in primates).

Neurons highly synchronized at 60 Hz were observed in the LGN (Saleem et al., 2017) and the retina (Storchi et al., 2017), suggesting a subcortical origin for such cortical narrow band. Recent modeling works reproducing in detail the structure of the mouse visual cortex (Arkhipov et al., 2018; Billeh et al., 2020) did not specifically address this issue. Overall, the function of the mouse γ narrow band in the V1 and the mechanisms underlying its modulation still need to be properly clarified.

To address this issue, we stimulated head-fixed awake mice (*n* = 12) with alternating gratings of different contrasts and performed a spectral analysis of the resulting V1 LFPs. We showed that narrow and broad γ band (BB) not only have opposite but even complementary sensitivity range. Simulations with recurrent excitatory-inhibitory spiking network accurately reproduced the behavior of both narrow and BB, supporting the origin of the former might be because of a periodic component embedded within the thalamic input.

## Materials and Methods

### Experimental design

#### Mice

Experiments were conducted in accordance with the European Community Directive 2010/63/EU and were approved by the Italian Ministry of Health. Animals were housed in a 12/12 h light/dark cycle with food and water available *ad libitum*. Adult (four to six weeks old) C57BL/6J female mice (*n* = 12) were used in all experiments until contrast level equal 50; only seven of them were instead employed for the maximum contrast level (K = 90) experiments.

#### Recording implant

Animals (*n* = 12) were chronically implanted with a custom-made aluminum head post, and a rectangular recording chamber (2 × 1.5 mm) of dental cement (Ivo-clar Vivadent Inc.) was built over the V1 (i.e., between 0 and 1.5 mm anterior and between 1.5 and 3.5 lateral to the λ suture) leaving the skull intact. A ground electrode was placed over the cerebellum. The electrode was connected to a pin socket and secured to the skull by acrylic dental cement. Surgery was conducted under deep avertin anesthesia (7 ml/kg; 20% solution in saline, i.p.; Sigma-Aldrich). Animals were then allowed to recover for 3 d. Following recovery, animals were habituated for 3 d to the head fixation apparatus. A craniotomy overlying V1 was performed 24 h before the first recording session. To preserve the cortical surface, the recording chamber was filled with a layer of agar (Sigma-Aldrich) and the silicone elastomer Kwik (World Precision Instrument) as a protective cap. In order to discard nonvisually evoked neural confounding, animals were restrained from moving while in the head fixation apparatus.

#### Extracellular recordings in awake mice

Recordings were performed on awake mice (*n* = 12). Mice were carefully placed in the head fixation apparatus. After removing the protective cap, the recording chamber was filled with sterile saline solution (0.9%) to preserve and moisten the tissue.

**Figure 1.**
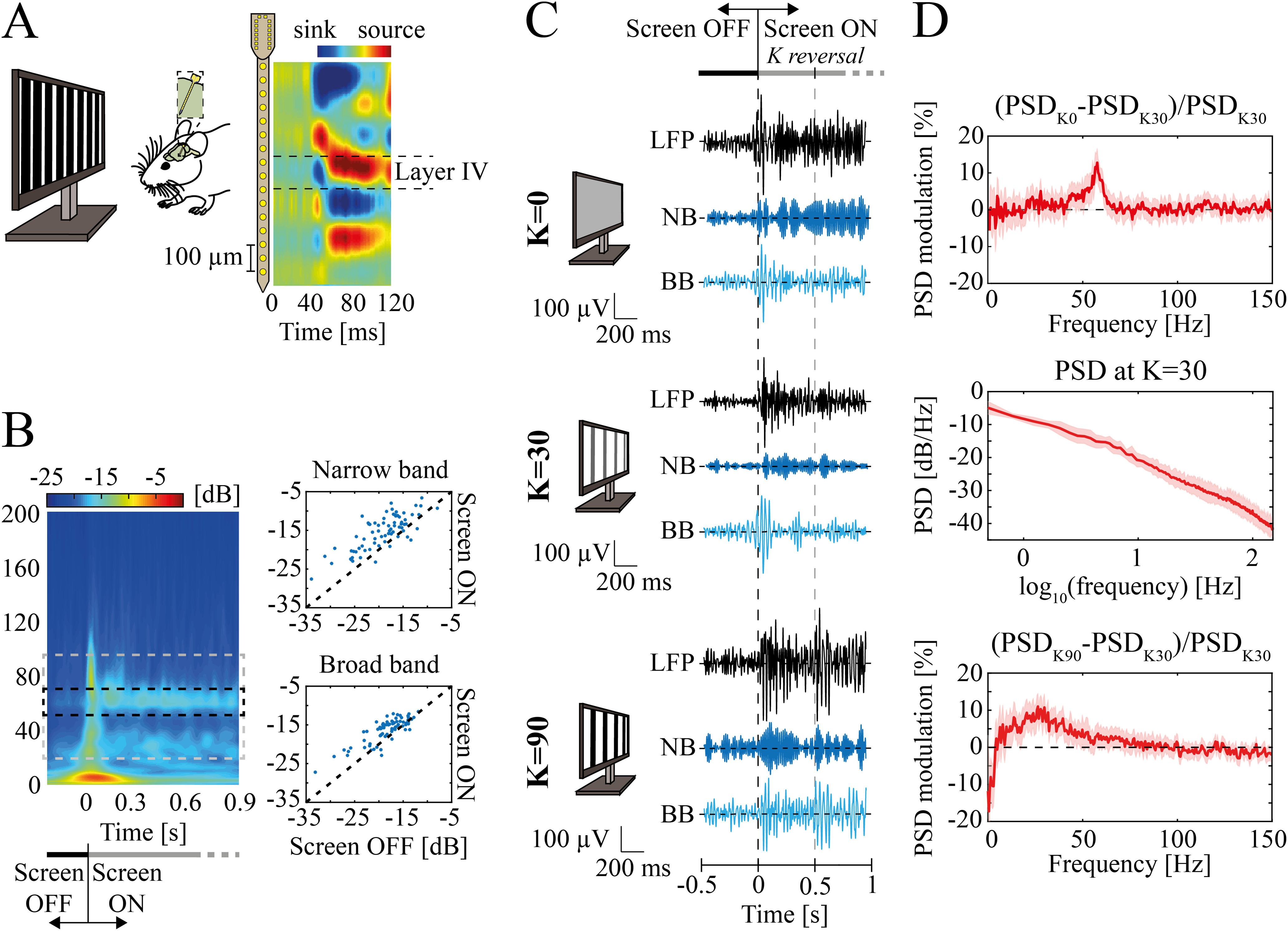
Experimental setup and data. ***A***, left, Representative scheme of the experimental design. Square-wave 1-Hz alternating gratings at different contrast levels were used for visual stimulation. A linear 16-channels probe (with 50-μm spacing between electrodes) was inserted into the mouse V1. Right, Mean across animals of current source densities (CSDs) aligned by the earliest current sink. ***B***, left, Mean scalogram for contrast equal to 0 (K=0) within −200 to 900 ms around screen onset (for K = 0). The dashed rectangle depicts the frequency bands ranges: narrow band (NB, middle, black) and broad band (BB, gray). Right, Scalogram magnitude comparison between screen OFF (from −200 to 0 ms) and screen ON condition (from 0 to 900 ms) in high NB (top) and BB (bottom). Each dot represents one stimulus presentation for one mouse (*n* = 12 animals, 70 experimental points overall). Statistical differences were accounted for by the Wilcoxon’s matched pairs signed-rank test. For both γ bands, *p* values were far less than 0.001. ***C***, Examples of filtered local field potential (LFP) recorded in mice V1 while viewing a uniform gray screen (top), or alternating gratings at contrast K = 30 (middle) and K = 90 (bottom). The examples are reported between [−0.5; 1] s around screen onset. Examples were filtered between (1) 10–100 Hz (black traces) just for representative purposes; (2) 45–65 Hz to display the NB; (3) from 20 to 45 Hz and from 65 to 90 Hz to display the BB range. Dashed lines indicate screen onset (black) and the first contrast reversal (gray). Monitors’ sketches schematically represent visual contrast. ***D***, LFP modulation of minimal contrast (i.e., K = 0; top) and maximal contrast (i.e., K = 90; bottom) with respect to the power spectral density (PSD) at K = 30 (middle). Modulation is defined as the difference between the power of a frequency at a given contrast level (K = 0 or K = 90 in this case) with the power at reference contrast K = 30, normalized to the latter power. Shaded regions indicate standard error of the mean (SEM).

A NeuroNexus Technologies 16-channel silicon probe (Fig. 1*A*) with a single-shank (A1x16-3 mm-50-177) was mounted on a three-axis motorized micromanipulator and slowly lowered into the visual cortex (in the central region of the recording chamber) till the depth of 1000 μm. Before the beginning of the recording, the electrode was allowed to settle for ∼5 min. The neurophysiological data were continuously recorded using a 16-channel Omniplex system (Plexon). At the end of the extracellular recording session, the recording chamber was covered with the protective cap as described above.

Each of the twelve animals underwent two recording sessions on two different days (one per day). Each experimental session was made up of at least two visual contrast sweeps. Specifically, each sweep consisted in presenting the mice with five contrast levels [0 10 20 30 50] % (*n* = 5 animals) or six contrast levels [0 10 20 30 50 90] % (*n* = 7 animals) in increasing order. Each visual stimulus consisted of 1-Hz alternating gratings presented for 30 s at a single contrast level to the head-fixed animals (see below, Visual stimuli). Overall, for each level of contrast below K = 90 we have two recording sessions × (≥2 sweeps) × 12 animals ≥ 48 trials of 30 s each. For k = 90, we have two recording sessions × (≥2 sweeps) × 7 animals ≥ 28 trials of 30 s each. Few recordings were removed because of artifacts. The actual numbers of trials used for every analysis is reported in the main text.

All the electrophysiological data were processed using custom-written MATLAB codes (The MathWorks) on a Windows 10 Home Dell XPS 15 laptop. The extracellular signals were sampled at 1 kHz and lowpass filtered at 200 Hz (filter type: Bessel, four poles).

#### Visual stimuli

Visual stimuli were computer-generated using the MATLAB Psychophysics Toolbox with γ correction and presented on a display (Sony; 40 × 9 × 30 cm; mean luminance 15 cd/m^2^) placed 25 cm from the head of the mouse (Fig. 1*A*), covering the center of the visual field. Extracellular signals were recorded in response to abrupt reversals (1 Hz) of vertical square-wave gratings (spatial frequency, 0.06 c/°; contrast levels adopted: [0 10 20 30 50 90] %). Signals were amplified (5000-fold), bandpass filtered (0.5–500 Hz), and fed into a computer for storage and analysis. For each recording, alternating gratings were presented for 30 s at a single contrast level to the head-fixed animals. To ensure consistency across animals (*n* = 12 for K ≤ 50 and *n* = 7 for K = 90), the orientation of the gratings was always vertical. It is worth mentioning that there is little evidence for columnar organization of orientation-selective neurons in the V1 of the mouse (Ko et al., 2011).

### Neurophysiological data analysis

#### Current source density (CSD) analysis and Layer IV identification

For each channel, visual evoked potential waveforms in response to contrast reversals were extracted from the LFPs by signal averaging. For each recording session, CSD was computed by applying a standard algorithm (according to the second spatial derivative estimate of the laminar LFP time series; Haberly and Shepherd, 1973; Freeman and Nicholson, 1975) along with the iCSD toolbox for MATLAB (Pettersen et al., 2006). A value of 0.3 S/m was taken as a measure of cortical conductivity (Gaussian filter: SD = 0.05 mm). We focused our analysis on Layer IV as it is the layer where sensory-induced γ oscillations are prominent (Welle and Contreras, 2016). Layer IV was identified in each recording session (two for each of the 12 mice) with the channel corresponding to the earliest current sink.

#### Spectral analysis

**Figure 2.**
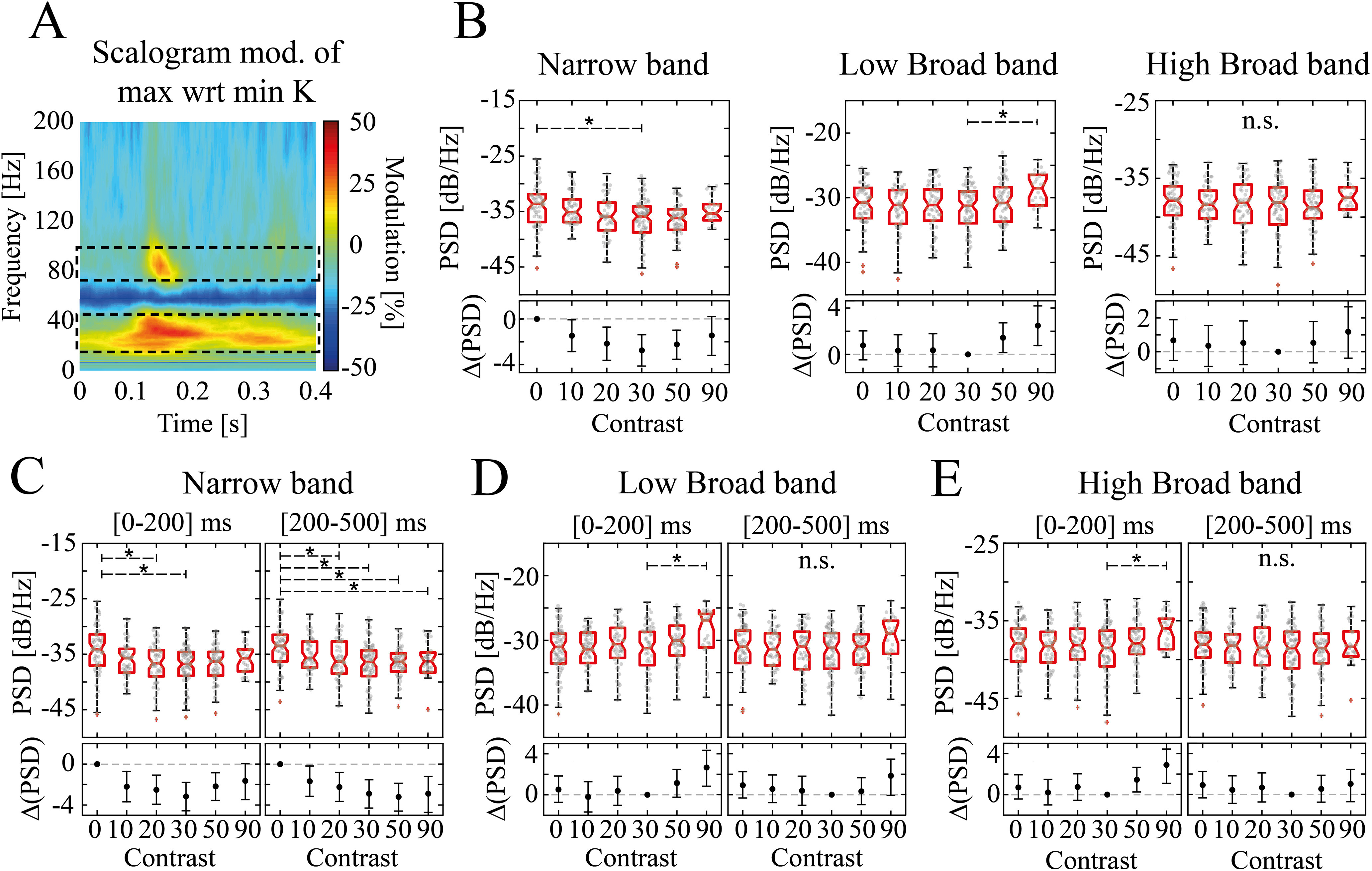
Narrow and broad band γ contrast-driven modulation in Layer IV of mouse V1. ***A***, K = 90 versus K = 0 modulation of LFP scalogram, pooled across all animals (*n* = 12 for all K < 90 and *n* = 7 for K = 90) and averaged across trials (in this case meaning both contrast reversals and recordings). Dashed black rectangles indicate the broad band (BB) composed of two ranges: low BB ([45–65] Hz) and high BB ([65–90] Hz). These two bands are separated by the narrow band (NB, [45–65] Hz). ***B***, PSD of NB (left), low BB (middle), high BB (right) as a function of visual contrast pooled across all animals and recordings over the whole [0–500] ms stimulation interval. On upper boxes, the horizontal red line indicates the median, bottom and top edge indicate the 25th and 75th percentiles. The whiskers extend to the most extreme data points not considered outliers (which are represented by red pluses). Asterisks indicate significant *post hoc* pairwise difference (K–W followed by Dunn’s test *p *<* *0.05). n.s. indicates nonsignificant statistical difference across contrasts. Individual experimental data points are indicated as gray circles. Lower boxes display the mean difference in PSD, ΔPSD, between a reference contrast (K = 0 for NB and K = 30 for the BB) and the other contrast levels. Black whiskers indicate the 95% confidence interval obtained through nonparametric bias-corrected bootstrap resampling (Ho et al., 2019). ***C–E***, Same as ***B*** for narrow band (***C***), low broad band (***D***), and high broad band (***E***) dividing the response interval in two windows: [0–200] ms (left) and [200–500] ms (right).

From extracellular recordings, we extracted LFPs by low pass filtering at 200 Hz (Fig. 1*C* for filtered LFP examples). LFPs were z-scored before spectral analysis. The power spectral density (PSD) of the z-scored LFPs was computed with the Fast Fourier Transform via the Welch method (*pwelch* function in MATLAB), dividing the time window under investigation into subwindows of 500 ms with 50% overlap. The only exception was when investigating the temporal structure of narrow and broad band PSD (Fig. 2*C*): z-scored LFPs were segmented in consecutive temporal windows after each contrast reversal. PSDs were, consequently, estimated independently for each of them and averaged across trials. Given the short duration of the time windows (200 and 300 ms) the Welch method was applied with no subwindow.

The PSDs were converted in decibels: PSD_dB_ = 10log_10_ (PSD).

Since the contrast *K *=* *30 minimized both narrow and broad band γ power (see Results), we expressed the spectral response at other contrasts as the spectral modulation relative to the response to this stimulus (Fig. 1*D*, top and bottom):

(1) $$PSD_{modulation}\left(K^{\prime }\right)=\frac{PSD_{K^{\prime }}-PSD_{K=30}}{PSD_{K=30}}.$$

Where *PSD_K_*_=30_ is the median LFP PSD across recordings and animals when contrast K = 30 is presented (Fig. 1*D*, middle) and *PSD_K'_* is the median LFP PSD across recordings and animals when contrast K = K’ is presented. The alternative option of computing the modulation relatively previsual baseline LFP led to similar results (data not shown).

**Figure 3.**
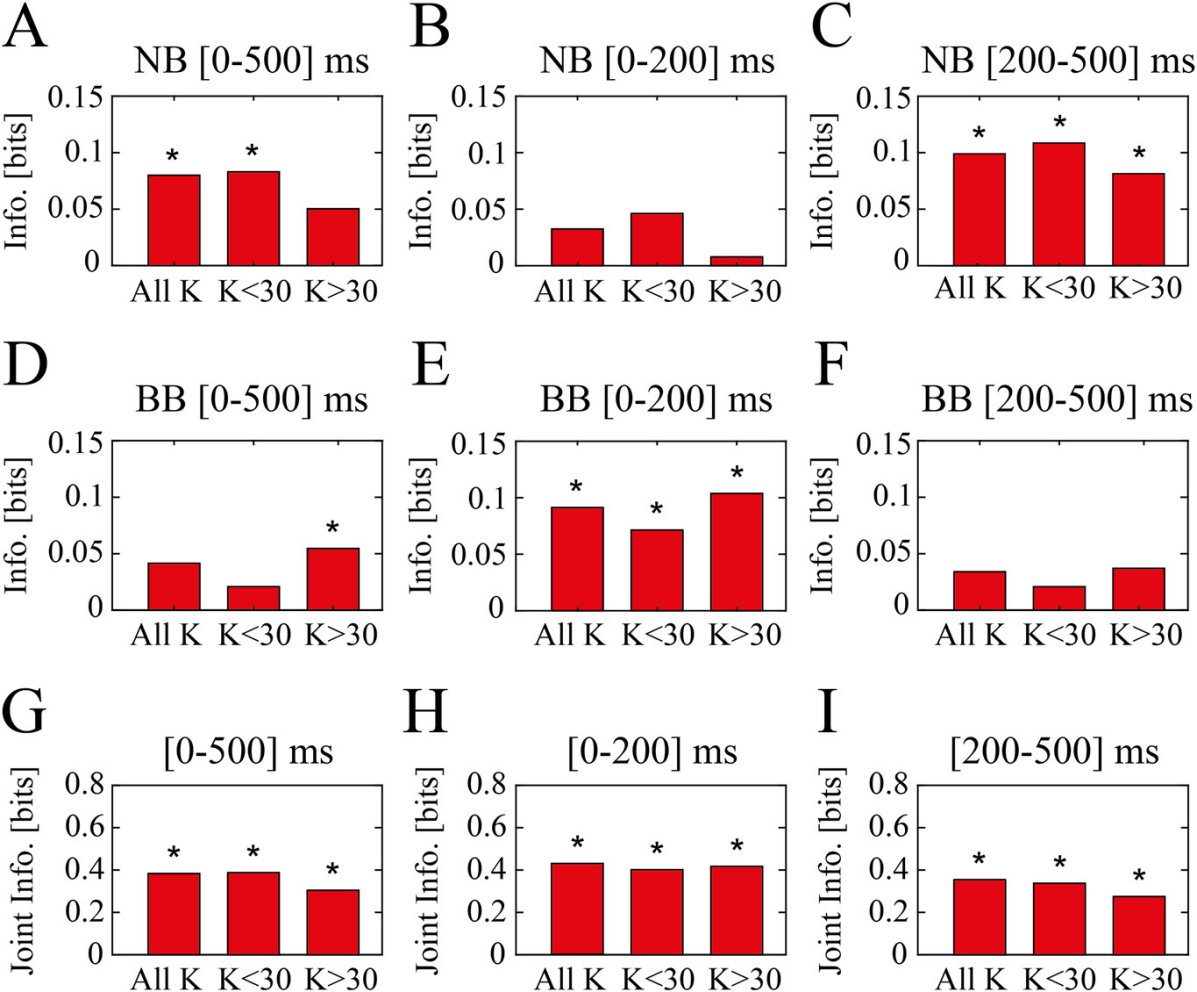
Information theory analysis reveal complementary encoding of visual contrast ranges. ***A***, Information carried by PSD modulation with respect to K = 30 of narrow band about all contrast levels (all K), low range of contrasts (K < 30), and high range of contrasts (K > 30). PSD modulation was considered for the whole inter-contrast-reversal interval ([0–500] ms). Asterisks indicate mutual information values exceeding the significance threshold (*p *<* *0.05; bootstrap test). ***B***, Same as ***A*** but considering only 200 ms following contrast reversal. ***C***, Same as ***A*** but considering the time window [200–500] ms following contrast reversal. ***D–F***, Same as ***A–C*** for broad band. ***G***, Joint mutual information carried by both narrow and broad band PSD modulation during the whole inter-contrast-reversal interval ([0–500] ms) about all contrast levels (all K), low range of contrasts (K < 30), and high range of contrasts (K > 30). ***H***, Same as ***G*** but considering only 200 ms following contrast reversal. ***I***, Same as ***G*** but considering a time window [200–500] ms following contrast reversal.

**Figure 4.**
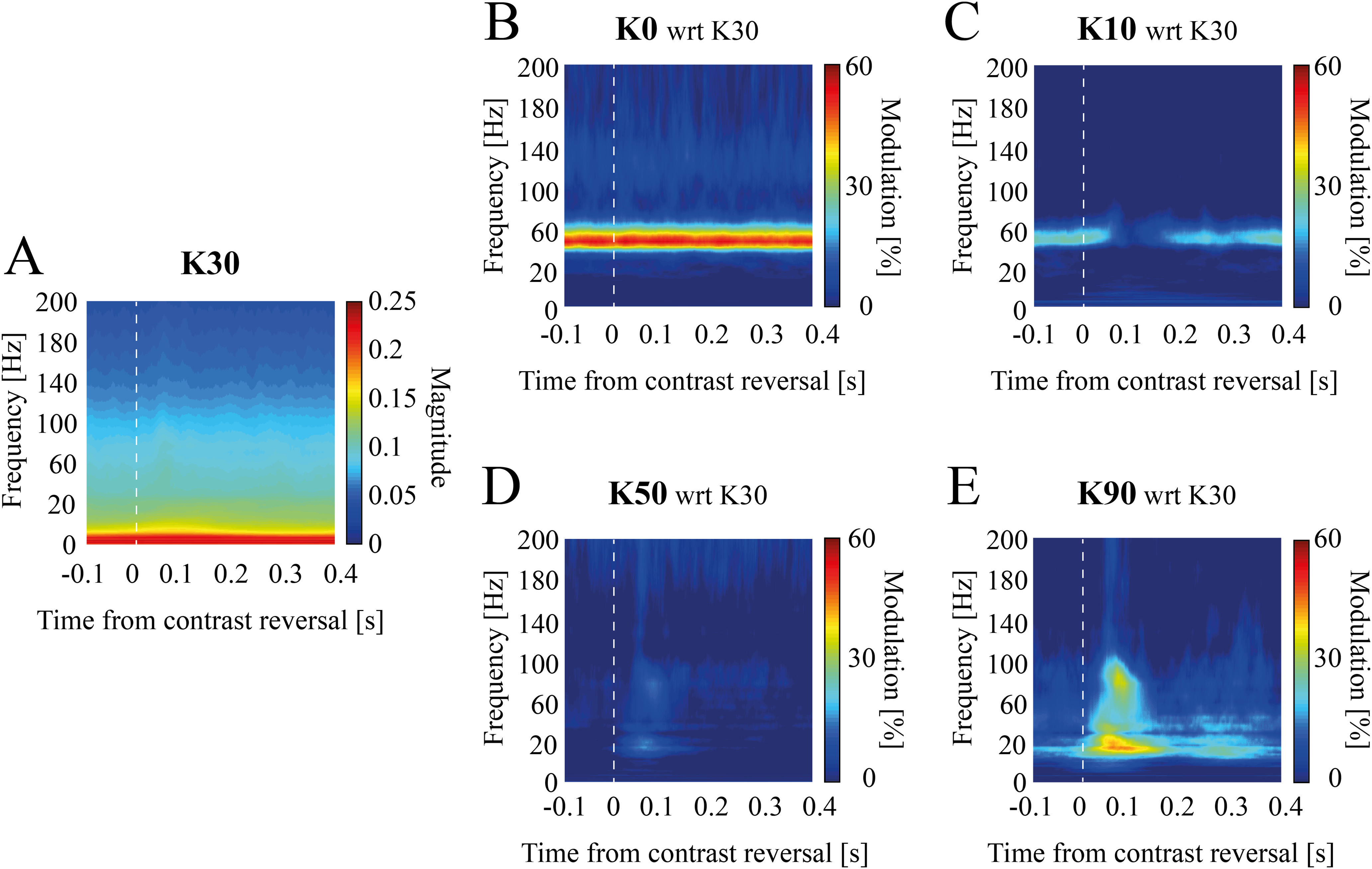
Temporal evolution of contrast-dependent spectral modulation in experimental data. Mean scalogram modulation with respect to K = 30 (***A***) for experimental data at K = 0 (***B***), K = 10 (***C***), K = 50 (***D***), and K = 90 (***E***). Scalograms were averaged [−100–400] ms around contrast reversal instants (see Materials and Methods).

We investigated the evolution in time of the LFP by means of wavelet analysis. LFP scalograms were computed using the continuous wavelet transform (*cwt* function in MATLAB), using the analytic Morse wavelet with symmetry parameter equal to three and the time-bandwidth product equal to 60. Scalograms were separately computed for each experimental recording and then split into 500-ms consecutive time windows: specifically, from −100 to 400 ms around every square-wave contrast reversal. We decided not to include in the analysis of the response to visual contrasts the LFPs evoked by the visual stimulus onset (i.e., the first 500 ms of the recorded LFP after stimulus onset also called screen onset hereafter) since it induced flash-like responses as the prestimulus consisted in a dark screen (Fig. 1*B*). As for the PSD modulation analysis (Fig. 2 and Fig. 3), the mean scalogram of the LFPs at K = 30 was the reference time-frequency map on relatively to which we computed the modulation of the other contrast levels (Fig. 4*A*). The scalogram modulation was computed by averaging the modulated time-frequency maps of every recording at a given contrast level (segmented in 500-ms windows around the grating reversal as described above). We computed then the median of the modulated scalogram of every recording within the narrow or the broad band to compute the time evolutions of these two γ bands.

To define the broad and narrow band frequency limits, we computed the modulation of the mean scalogram across trials and animals of K = 90 with respect to K = 0 (Fig. 2*A*). We defined broad band (BB) the set of frequencies for which the modulation exceeded the 20%, i.e., the two disjoint ranges [20–45] and [65–95] Hz. We defined NB, the γ band interval between these two ranges, i.e., [45–65] Hz.

Next, we evaluated the onset latency of narrow band activation. We computed, for every recording, the onset time of the intertrial median NB modulation amplitude (Bartoli et al., 2019). First, for each NB signal, we marked the first time point at which the NB amplitude exceeded a threshold (<25th percentile for at least 40 ms in the time window between −100 and 400 ms; ∼40% of the trials were discarded as they did not meet this criterion). Next, a 200-ms wide window was extracted around that time point (100 ms before and 100 after). This window was segmented into 50-ms bins with 80% overlap and a linear regression was fit to each bin. The first time point of the bin with the highest slope and smallest residual error was defined as the onset of the narrowband γ oscillations.

The same procedure was repeated to estimate broad band onset latency, with a threshold of >75th percentile because of the opposite polarity of the modulation.

#### Spectral information analysis

To determine how well the PSD modulation of the narrow band encoded the contrast level of visual stimuli, we computed the mutual information (Shannon, 1948) I(K; NB_mod_) carried by the narrow band PSD modulation with respect to K = 30 throughout the whole intercontrast-reversal interval, NB_mod_, about the set of contrasts K:

(2) $$I\left(K;NB_{mod}\right)=\underset{k}{\sum }P\left(k\right)\underset{nb_{mod}}{\sum }P\left(nb_{mod},k\right)log_{2}\frac{P\left(nb_{mod},k\right)}{P\left(nb_{mod}\right)},$$

where *P*(*K*) is the probability of contrast K to be presented, *P*(*nb_mod_*) is the probability distribution of the narrow band modulation over all the contrasts, and *P*(*nb_mod_*, *k*) is the probability of the NB modulation *nb_mod_* to be observed when contrast stimulus K is presented. The probabilities, both marginal and conditional, were computed by discretizing the power modulations into 10 equipopulated bins. Specifically, the discretization boundaries were chosen so that each bin contains the same number of elements. This discretization choice, in place of an “equal-width” binning procedure, ensures robustness against outlier and maximizes the response entropy (Timme and Lapish, 2018). The same analysis was performed for the broad band. Because of the small number of trials for single animals, probabilities were estimated over all trials and recordings.

Similarly, we also computed the mutual information carried by NB and BB when considering the PSD modulation within 200 ms or from 200 to 500 ms following contrast reversal.

Limited dataset bias was accounted for by applying the Panzeri–Treves correction (Panzeri and Treves, 1996).

We further investigated the amount of information carried by the two γ bands about low or high contrast levels (low: K < 30 and high: K > 30). Low contrast information was computed as described above, after random permutation of high contrast-response association (average over 500 permutations), and vice-versa. In this case the bias was accounted for by applying bootstrap correction and quadratic extrapolation (Panzeri and Treves, 1996; Magri et al., 2009).

Finally, the synergistic contribution in the mutual information when jointly considering both narrow and broadband γ modulation was computed as:

(3) $$Syn\left(K;NB_{K},BB_{K}\right)=I\left(K;NB_{K},BB_{K}\right)-\left[I\left(K;NB_{K}\right)+I\left(K;BB_{K}\right)\right]+\;Red\left(I\left(K;NB_{K}\right),I\left(K;BB_{K}\right)\right),$$

where *I*(*K*; *NB_K_*, *BB_K_*) is the joint information carried by both narrow and broad band modulation, *I* (*K; NB_K_*) + *I*(*K; NB_K_*) is the sum of the information carried about visual contrast level by narrow and broad band γ modulation, respectively, and *Red* [*I* (*K*; *NB_K_*), *I* (*K*; *BB_K_*)] is the amount of redundancy (i.e., the amount of information overlap existing between the two γ bands power about the stimulus levels).

We have employed as a measure of redundancy the one proposed by Williams and Beer (2010), namely the minimum information: redundancy is expressed as the expected value of the minimum information that any response variable carries individually about each one of the stimuli.

(4) $$Red\left(I\left(K;NB_{K}\right),I\left(K;BB_{K}\right)\right)=\underset{k}{\sum }p\left(k\right)*\min\left\{I\left(K=k,NB_{k}\right);I\left(K=k,BB_{k}\right)\right\}.$$

Significant information (*p *=* *0.05) was estimated using the 95th percentile of bootstrap information. All information quantities were computed in MATLAB with Information Breakdown Toolbox (Magri et al., 2009).

### Network model

#### Spiking network model of mice V1

**Figure 5.**
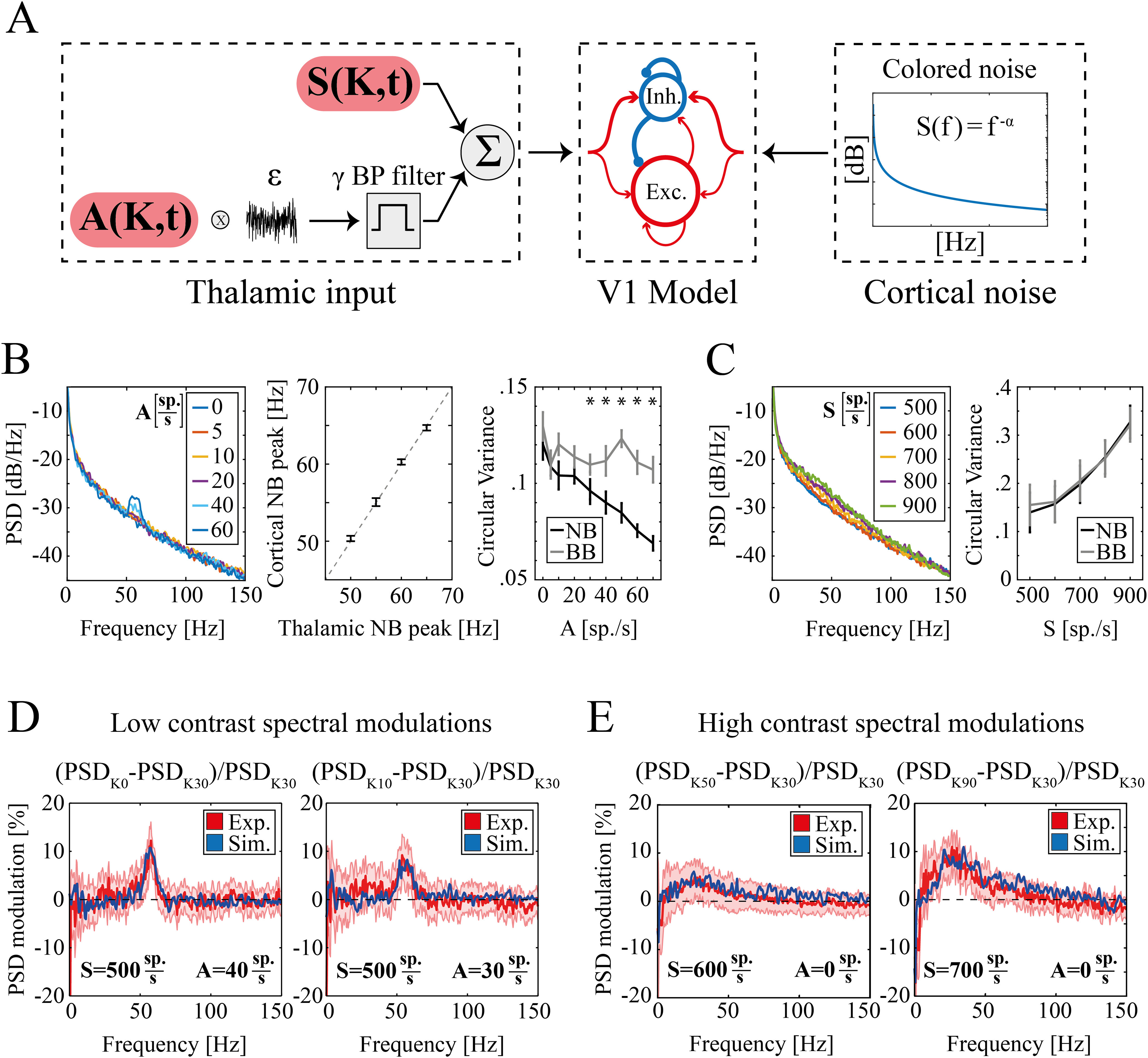
Experimental and simulated spectral modulation at different visual contrasts. ***A***, Schematic representation of the primary visual cortex (V1) model network. From left to right, (1) Thalamic inputs; sustained component S(K,t) (top) and periodic component of amplitude A(K,t) (bottom); (2) sparse LIF network of excitatory (*n* = 4000, red) and inhibitory neurons (*n* = 1000, blue). The size of the arrows represents schematically the strength of single synapses. In addition to recurrent interactions, both populations receive external excitatory inputs; (3) colored noise (see Materials and Methods) modeling ongoing unstructured cortical inputs. ***B***, left, power spectral density (PSD) of simulated LFPs as a function of the amplitude of periodic thalamic input A (legend in the inset indicate A values). Middle, Cortical NB frequency peak as a function of the corresponding frequency peak of the thalamic NB. Dashed gray line indicates the identity line. Right, Circular variance of the phase difference between the simulated LFP and the thalamic input within the NB (black line) and within the BB (gray line) as a function of the amplitude of periodic thalamic input A. Asterisks indicate levels of input for which NB circular variance is significantly less than BB (*p *=* *0.002). ***C***, left, PSD of simulated LFPs for increasing levels of the sustained component of the thalamic input S (legend in the inset indicate S values). Right, Circular variance as a function of the amplitude of the sustained thalamic input S for the phase difference between the simulated LFP and the thalamic input within the NB (black line) and the BB (gray line). Circular variance was significantly modulated by the sustained component of the thalamic input S both within the NB (K–W test *p *≪* *0.001) and the BB (K–W test *p *≪* *0.001). Circular variance was likewise modulated within these two bands [two-way ANOVA: *p *=* *0.99 for the interaction between the value of sustained thalamic input (S) and the frequency band (NB or BB)]. ***D***, Modulation of LFP PSD for K = 0 (left) and K = 10 (right) with respect to K = 30 in experimental (red) and simulated (blue) data. The red shaded area represents SEM of experimental data across animals and recordings. In both simulations, sustained thalamic input was set to S = 500 sp./s, while the periodic input was set to A = 40 sp./s for K = 0 (left) and A = 30 sp./s for K = 10 (right). ***E***, Modulation of LFP PSD for K = 50 (left) and K = 90 (right) with respect to K = 30 in experimental (red) and simulated (blue) data. The red shaded area represents SEM of experimental data across animals and recordings. In both simulations, periodic thalamic input was set to A = 0 sp./s, while the sustained input was S = 600 sp./s for K = 50 (left) and S = 700 sp./s for K = 90 (right).

The simulated network is composed of *N* = 5000 leaky integrate and fire (LIF) neurons (Tuckwell, 1988): 80% excitatory neurons with AMPA-like synapses, and 20% inhibitory neurons with GABA-like synapses (Braitenberg and Schüz, 1991). The network is sparse and random, the connection probability between any directed pair of cells being 0.2 (Sjöström et al., 2001; Holmgren et al., 2003; Fig. 5*A*, middle).

The membrane potential *V^k^* of each neuron *k* evolves according to (Brunel and Wang, 2003):

(5) $$\tau_{m}\frac{dV^{k}\left(t\right)}{dt}=-V^{k}\left(t\right)+\frac{I_{tot}^{k}\left(t\right)}{g_{leak}},$$

where *τ_m_* is the membrane time constant, *g_leak_* is the leak membrane conductance (see Table 1 for values) and $I_{tot}^{k}\left(t\right)$ is the total synaptic input current. The latter was given by the sum of all the synaptic inputs entering the k-th neuron:

(6) $$I_{tot}^{k}\left(t\right)=\underset{j\in AMPA}{\sum }C_{jk}I_{AMPA}^{k}\left(t\right)+\underset{j\in GABA}{\sum }C_{jk}I_{GABA}^{k}\left(t\right)+I_{EXT}^{k}\left(t\right).$$

**Table 1:** Synaptic parameters

	GABA on inhibitory	GABA on excitatory	AMPA_recurrent_ on inhibitory	AMPA_recurrent_ on excitatory	AMPA_external_ on inhibitory	AMPA_external_ on excitatory
*g_syn_* (nS)	2.700	2.010	0.233	0.178	0.317	0.234
*τ_l_* (ms)	1	1	2	2	2	2
*τ_r_* (ms)	1	1	0.2	0.4	0.2	0.4
*τ_d_* (ms)	5	5	1.25	2.25	1.25	2.25

Where *C_jk_* ≠ 0 if j projects to k, and $I_{AMPA}^{k}\left(t\right)$, $I_{GABA}^{k}\left(t\right)$, $I_{EXT}^{k}\left(t\right)$ the different synaptic inputs entering the k-th neuron from recurrent AMPA, GABA, and external AMPA synapses, respectively.

The synaptic inputs currents were modeled as:

(7) $$I_{syn}^{k}\left(t\right)=g_{syn}s_{syn}\left(t\right)\left(V^{k}\left(t\right)-V_{syn}\right),$$

where *g_syn_* are the synaptic conductances (Markram et al., 1997; Gupta et al., 2000; Bartos et al., 2001, 2002; see Table 1), and *V_syn_* are the reversal potential of the synapses (V_GABA_ = −80 mV and V_AMPA_ = 0 mV). The function *s_syn_*(*t*) described the time course of the synaptic currents and depends on both the synapse type and on the kind of neuron receiving the input. Specifically, every time a presynaptic spike occurred at time t*, *s_syn_* (*t*) of the postsynaptic neuron was incremented by an amount described by a delayed difference of exponentials (Brunel and Wang, 2003):

(8) $$\Delta s_{syn}\left(t\right)=\frac{\tau_{m}}{\tau_{d}-\tau_{r}}\left[\exp\left(-\frac{t-\tau_{l}-t^{*}}{\tau_{d}}\right)-\exp\left(-\frac{t-\tau_{l}-t^{*}}{\tau_{r}}\right)\right],$$

where the latency *τ_l_*, the rise time *τ_r_* and the decay time *τ_d_* are listed in Table 1 for each synapse type (Xiang et al., 1998; Zhou and Hablitz, 1998; Angulo et al., 1999; Gupta, 2000; Kraushaar and Jonas, 2000; Bartos et al., 2001).

The LFP of the simulated network was estimated as the sum of the absolute value of the GABA and AMPA currents (both external and recurrent) that enter all excitatory neurons (Mazzoni et al., 2015). Simulated LFP was analyzed with the same procedures followed for experimental LFP (see above).

#### External inputs to the simulated network

For all neurons the external input $I_{EXT}^{k}\left(t\right)$ is the sum of two terms: a noisy excitatory external input representing the activity from thalamocortical afferents (Fig. 5*A*, left) and colored noise mimicking stimulus-unspecific cortical activity (Fig. 5*A*, right). This simulated external input was implemented as a series of spike times that activated excitatory synapses with the same kinetics as recurrent AMPA synapses, but different strengths (Table 1). These synapses were activated by independent realizations of random Poisson spike trains, with a time-varying rate identical for all neurons. Based on experimental recordings from mice LGN (Saleem et al., 2017; McAfee et al., 2018), this time-variant rate was given by the superimposition of a constant component and a periodic term at γ frequency:

(9) $$v_{ext}\left(t\right)={\left[S\left(K,t\right)+A\left(K,t\right)\varepsilon_{\gamma }\left(t\right)+\vartheta_{n}n\left(t\right)\right]}_{+},$$

where *K* is the contrast level, *S*(*K*, *t*) the sustained input rate, *A*(*K*, *t*) the amplitude of γ range filtered white constant noise *ε_γ_* (*t*), and *n*(*t*) is the colored noise. The first two terms represent thalamic inputs (Fig. 5*A*, left). Specifically, *ε_γ_*(*t*) was obtained by applying a third-order bandpass Butterworth filter of central frequency equal to 57 Hz and bandwidth equal to 10 Hz to white noise. The only exception was when investigating the relationship between cortical and thalamic NB peak frequency (Fig. 5*B*, middle). In this case, we modulated the central frequency of the oscillatory component of thalamic input from 50–65 Hz in three steps of 5 Hz each.

**Figure 6.**
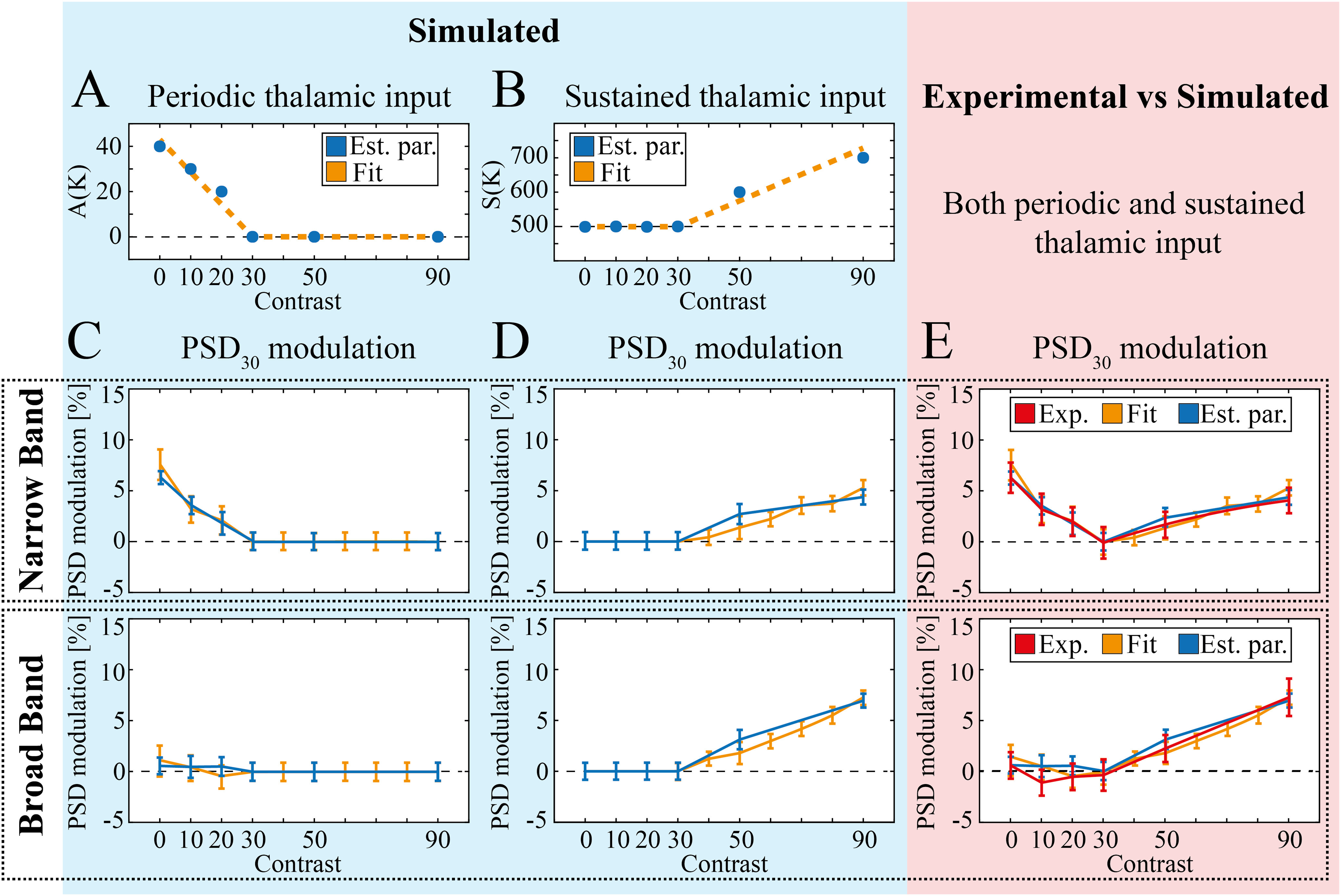
Simulation of NB and BB γ over the whole contrast range. ***A***, ***B***, Optimal amplitude of periodic (***A***) and sustained (***B***) thalamic input amplitude for each value of contrast K (blue markers) and corresponding fit (dashed orange line). ***C***, PSD_30_ modulation (see Materials and Methods) of NB (top) and BB (bottom) with fixed sustained input S = 500 sp./s and individual A(K) values shown in (***A***; blue line) and overall fit (orange line). Error bars indicate mean ± SEM here and in the following panels. ***D***, PSD_30_ modulation (see Materials and Methods) of NB (top) and BB (bottom) with sustained input S(K) values shown in ***B*** (blue line) or associated fit (orange line) and no periodic input. ***E***, Comparison of PSD_30_ modulation in NB (top) and BB (bottom) for experiments (red) and simulations with A(K) and S(K) values determined by local optimization (blue) or associated fit (orange).

The noise term *n*(*t*) is a z-scored colored noise, with the PSD following $S\left(f\right)=\frac{1}{f^{\alpha }}$, with *α* = 1.5, and an amplitude factor $\vartheta_{n}=0.4\frac{sp.}{ms}$. Both *ε_γ_*(*t*) and *n*(*t*) were independently generated at every simulation. […]_+_ is a threshold-linear function, [*x*]_+_ = x if *x *>* *0, [*x*]_+_ = 0 otherwise, to avoid a negative number of spikes which could arise because of the noise terms. In the first part of our work (Figs. 5, 6), we set the external input parameters to be time-invariant, i.e., *A*(*K*) and *S*(*K*) with a value for each K reported in Table 2 (see parameter selection).

**Table 2:** Thalamic input parameters

	K = 0	K = 10	K = 20	K = 30	K = 50	K = 90
*A*(*K*) (sp./s)	40	30	20	0	0	0
*S*(*K*) (sp./s)	500	500	500	500	600	700
*A*_0_(*K*) (sp./s)	40	30	20	0	0	0
*α*(*K*) (sp./s)	0	30	20	0	0	0
*S*_0_(*K*) (sp./s)	500	500	500	500	500	600
*β*(*K*) (sp./s)	0	0	0	0	250	200

In particular, setting $A=0\frac{sp.}{s}$ and $S=500\frac{sp.}{s}$ the PSD of the simulated LFPs closely matched the reference experimental spectrum for K = 30 (Fig. 1*D*, middle; reduced χ^2^ between experimental and simulated LFPs PSD $X_{r}^{2}=0.13$). The spectral modulations of the other simulated contrast levels were consequently assessed using this median LFP PSD as a baseline, analogously to the approach adopted for the experimental data.

The values of *A*(*K*) and *S*(*K*) across K were approximated with two piecewise linear functions (Fig. 6*A*,*B*):

(10) A(K)=(42.8−1.4K)(u(K)∗u(30−K))

and

(11) S(K)=(384.8+3.8K)u(K−30),

where *K* is the contrast level intended to be simulated and *u*(*K*) is the Heaviside step function: *u*(*K*)* *=* *0 for *u*(*K*)* *<* *0 and *u*(*K*)* *=* *1 for *u*(*K*)* *≥* *0.

**Figure 7.**
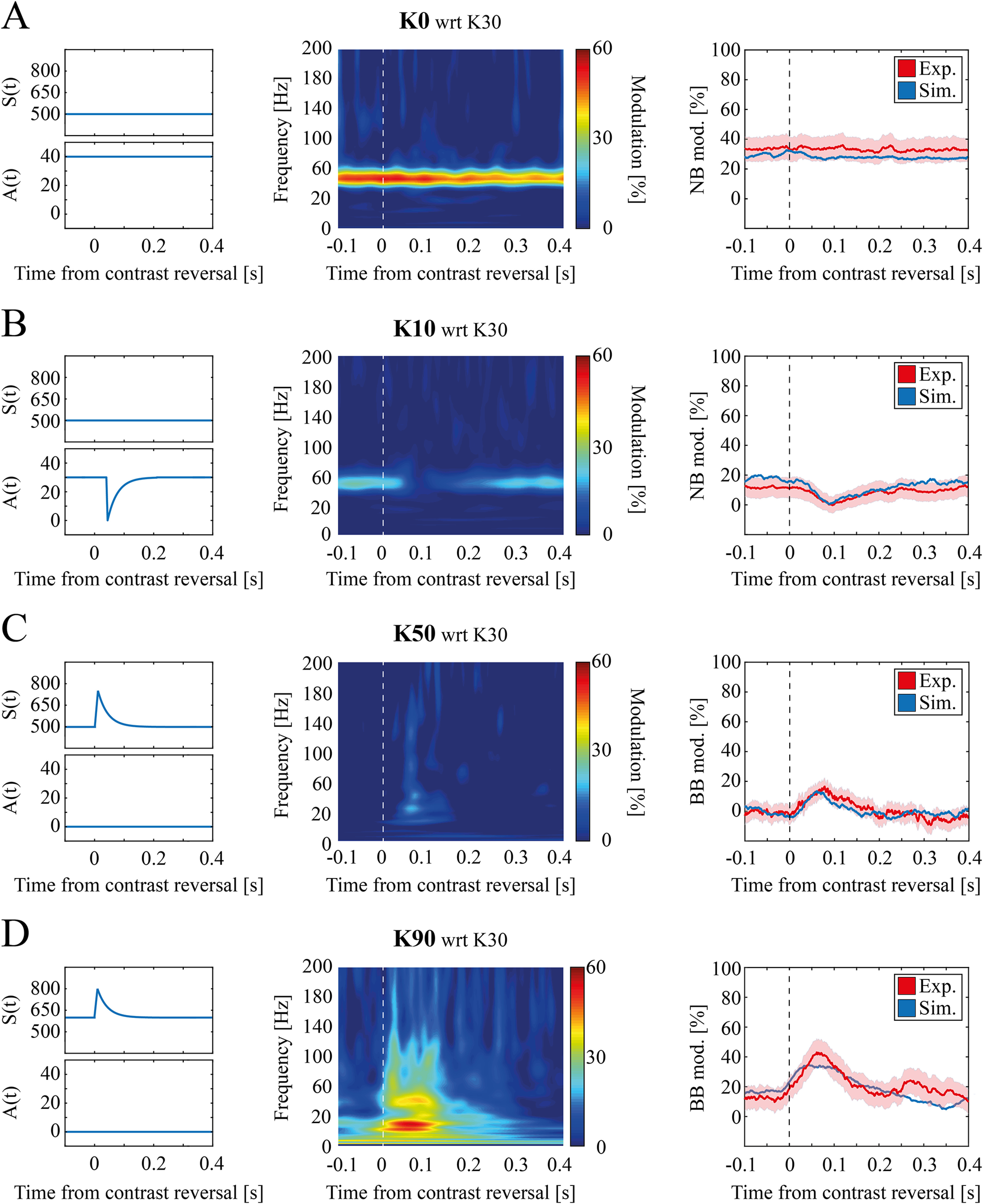
Temporal evolution of contrast-dependent modulation in simulated data. ***A***, left, Time course of thalamic input model parameters for K = 0. Middle, Scalogram mean modulation at K = 0 with respect to K = 30 for simulated data. Right, Time evolution of narrow band modulation for experimental (red) and simulated (blue) data (shading reflects SEM). ***B***, Same as ***A*** for K = 10. ***C***, left, Time course of thalamic input model parameters for K = 50. Middle, Scalogram mean modulation at K = 50 with respect to K = 30 for simulated data. Right, Time evolution of broad band modulation for experimental (red) and simulated (blue) data (shading reflects SEM). ***D***, Same as ***C*** for K = 90.

In the second part of the work (Fig. 7), we also took into account the evolution in time of the external input parameters. As for the amplitude of the γ range filtered white noise, i.e., *A*(*K*, *t*), we defined it as a function of the time of grating reversal (see above, Visual stimuli):

(12) $$A\left(K,t\right)=A_{0}\left(K\right)-\alpha \left(K\right)f\left(t-t^{*}-Dt\right)$$

with

(13) $$f\left(t\right)=\{\begin{array}{c} \frac{t}{10}for\;t<10ms\\ \text{exp }\left(-25\left(t-10\right)\right)for\;t\geq 10ms \end{array},$$

where *A*_0_(*K*) is the baseline value, *α*(*K*) is the amplitude of the reversal-driven modulation *f*(*t*), t* is the time of grating reversal and Dt is 40 ms to mimic the latency of the narrowband observed experimentally. The finite rise time was estimated to ∼10 ms (Fig. 7*C*,*D*) and for the sake of simplicity was approximated with a linear growth. The decay time was defined to reproduce the quick decay observed experimentally, with the transient effects ending after ∼200 ms (Fig. 7). The values of *A*_0_(*K*) and *α*(*K*) are reported in Table 2 (see inputs parameter selection).

Similarly, we set the sustained thalamic input *S*(*K*, *t*) to:

(14) $$S\left(K,t\right)=S_{0}\left(K\right)+\beta \left(K\right)f\left(t-t^{*}\right),$$

where *S*_0_(*K*) is an additive constant and *β*(*K*) is the amplitude of the same reversal-driven modulation *f*(*t*) defined in Equation 13. The obtained time course of the thalamocortical afferents spike rate qualitatively matches the one observed experimentally by McAfee et al. (2018). The values of *S*_0_(*K*) and *β*(*K*) are reported in Table 2 (see below, Input parameters selection).

#### Input parameters selection

Starting from experimental observations (Figs. 1-4) we defined the synaptic inputs A(K) and S(K) to be complementary. Setting $A\left(K=30\right)=0\frac{sp.}{s}$ and $S\left(K=30\right)=500\frac{sp.}{s}$ the resulting synthetic LFP spectra closely matched experimental results (see above). For all K > 30 A(K) was set to be zero and for all values K < 30 S(K) was set to be $500\frac{sp.}{s}$. To find the optimal parameters A(K) for K = [0, 10, 20] we simulated the LFP generated by inputs with values of A(K) ranging from 0 to 100 sp./s in steps of 10 sp./ms. For each input, we estimated the spectral modulation relative to K = 30 and we estimated the agreement with the experimental LFP spectral modulation as follows:

(15) $$X_{r}^{2}=\frac{1}{F}\overset{F}{\underset{f}{\sum }}\frac{{\left(mod^{sim}\left(f,S,A\right)-mod^{exp}\left(f,K\right)\right)}^{2}}{{\left(\sigma_{mod}^{exp}\left(f,K\right)\right)}^{2}},$$

where F is the total number of frequencies, *mod^sim^*(*f*, *S*, *A*) is the median modulation across simulations of the LFPs when setting the external input parameters to *S* and *A* (see Eq. 9), and *mod^exp^* (*f*, *K*) and $\sigma_{mod}^{exp}\left(f,K\right)$ are the median and the standard deviation of the experimental LFPs modulation at a given contrast *K* across animals and trials. For each K, the value of A(K) minimizing this measure was selected (see Table 2).

We set the optimal values of *S*(*K*) for K = [50, 90] (see Table 2) in a similar way, simulating the LFP generated by inputs in which *S*(*K*) varied from 500 to 1000 sp./s in steps of 100 sp./s.

Similarly, as we found no significant time structure in the experimental scalogram at K = 30 (Fig. 4*A*), for the time-variant model we set *S*_0_(*K *=* *30) = 500 sp./s, *A*_0_ (*K *=* *30) = *α*(K = 30) = *β*(K = 30) = 0 sp./s. As we have experimentally observed narrow and broad band to be complementary modulated in two different contrast ranges (Fig. 4), for K < 30 we set *β*(*K*)* *=* *0 sp./s and *S*_0_(*K*)* *=* *500 sp./s, whereas for K > 30 we set *α*(*K*) = *A*_0_ (*K*)* *=* *0 sp./s. Therefore, to find the optimal values of *S*_0_(*K*) and *β*(*K*) parameters for K = [50,90], we simulated the LFP generated by inputs with values of *S*_0_(*K*) ranging from 500 to 1000 sp./s in steps of 100 sp./ms and of *β*(*K*) ranging from 0 to 400 sp./s in eight uniform steps. For each input pair combination, we, therefore, computed the scalogram modulation relative to K = 30, we extracted the BB time evolution and we estimated the agreement with the experimental LFP scalogram modulation as follows:

(16) $$X_{r}^{2}=\frac{1}{T}\overset{T}{\underset{t}{\sum }}\frac{{\left(mod_{BB}^{sim}\left(t,S_{0},\beta \right)-mod_{BB}^{exp}\left(t,K\right)\right)}^{2}}{{\left(\sigma_{mod_{BB}}^{exp}\left(t,K\right)\right)}^{2}},$$

where T is the total number of time points, $mod_{BB}^{sim}\left(t,S_{0},\beta \right)$ is the median modulation of the simulated time course of the broad band γ range when setting the external input parameters to *S*_0_ and β, $mod_{BB}^{exp}\left(t,K\right)$ and $\sigma_{mod_{BB}}^{exp}\left(t,K\right)$ are the median and the variance modulation of the experimental time course of the broad band γ range at a given contrast level *K*.

Similarly, for K=[0,10,20] we simulated the LFP generated by inputs with values of *A*_0_(*K*) ranging from 0 to 100 sp./s in steps of 10 sp./ms and of *α*(K) ranging from 0 to *A*_0_(*K*) in steps of 10 sp./s. For each input pair combination, we, therefore, computed the scalogram modulation relative to K = 30, we extracted the NB time evolution and we estimated the agreement with the experimental LFP scalogram modulation as follows:

(17) $$X_{r}^{2}=\frac{1}{T}\overset{T}{\underset{t}{\sum }}\frac{{\left(mod_{NB}^{sim}\left(t,A_{0},\alpha \right)-mod_{NB}^{exp}\left(t,K\right)\right)}^{2}}{{\left(\sigma_{mod_{NB}}^{exp}\left(t,K\right)\right)}^{2}},$$

where T is the total number of time points, $mod_{NB}^{sim}\left(t,A_{0},\alpha \right)$ is the median modulation of the simulated time course of the narrow-band γ range when setting the external input parameters to *A*_0_ and α, $mod_{NB}^{exp}\left(t,K\right)$ and $\sigma_{mod_{NB}}^{exp}\left(t,K\right)$ are the median and the variance modulation of the experimental time course of the narrow-band γ range at a given contrast level K.

#### Phase analysis

To investigate the mechanisms underlying the spectral proprieties of the simulated LFPs, we investigated the relationship between the phase distribution of the LFPs and their corresponding thalamic inputs within the narrow and the BB (Fig. 5*B*,*C*). After filtering in the frequency band of interest, LFP and thalamic input phases were estimated via the Hilbert transform. The circular variance of the difference between these phase distributions was computed using the Circstat toolbox in MATLAB (Berens, 2009).

#### Simulations

Network simulations were performed using a finite difference integration scheme based on the second-order Runge–Kutta algorithm (Hansel et al., 1998; Shelley and Tao, 2001; Press, 2007) with time step Δ*t *=* *0.05 ms. To focus on stationary responses, the first 200 ms of every simulation were discarded.

Simulations with time-invariant inputs lasted 10 s and each set of inputs was presented 25 times with different noise terms *ε_γ_* (*t*) and *n*(*t*).

Simulations with time-dependent inputs lasted 2 s: during the first second, the input was set to the baseline values of $A=0\frac{sp.}{s}$ and $S=500\frac{sp.}{s}$; during the last second, instead, the input was determined by the level of contrast (see Table 2). Each set of input was presented 100 times with different noise terms *ε_γ_* (*t*) and *n*(*t*).

All simulations were conducted with custom made Python scripts within the Brian 2 simulator environment (Goodman, 2008; Stimberg et al., 2019) on a Windows 10 Home Dell XPS 15 laptop.

### Statistical analysis

Data processing and statistical analysis were carried on with custom-made MATLAB scripts and available third-party data analysis Toolboxes (e.g., for computing mutual information). We employed both custom scripts and built-in data analysis and statistical functions. For each statistical comparison throughout the text, we reported the statistical test and their *p* values; *p* values lower than 0.05 were considered significant. All results will be reported as median ± SEM unless otherwise stated. As for the results presented in Figure 2, we adopted the estimation graphics package presented in (Ho et al., 2019).

### Code accessibility

The code described in the paper used for simulating the LFPs is freely available online at https://github.com/nicolomeneghetti/mice-v1-narrow-broad-gamma-band. The code is available as Extended Data 1 contains the python code for running the simulations described in the manuscript. 

10.1523/ENEURO.0106-21.2021.ed1 Extended Data 1 Python code for running the simulations. The simulations employ the Brian2 simulation environment. Download Extended Data 1, TXT file.

## Results

### Broad and NBs in mice V1 display distinct sensitivity to contrast

We analyzed the spectral proprieties of Layer IV V1 LFPs recorded from awake mice presented with alternating gratings with different levels of visual contrast (see Materials and Methods; Fig. 1*A*). The animals (*n* = 12) were head-restrained during the recordings and were not allowed to move to avoid movement-driven modulations of the γ band (Niell and Stryker, 2010; Lee et al., 2014; Saleem et al., 2017). Coherently with previous results (Saleem et al., 2017), we found that the spectral modulation of the LFP with such stimuli was not uniform over the whole γ band [20–95] Hz, but it was characterized by the presence of a narrow (NB, [45–65] Hz) within a broad band (BB, [20–45] and [65–95] Hz) with distinct modulation proprieties. Both bands increased their power at stimulus onset (i.e., when turning the screen on with therefore luminance variation; Fig. 1*B*, Wilcoxon’s matched pairs signed rank test for 70 recordings across 12 animals *p *≪* *0.001 for both NB (top) and BB (bottom)].

The subsequent response (i.e., only to the reversal of contrast gratings) of NB was instead maximal for low contrasts (K = 0; Fig. 1*C*, top) while for BB was maximal at high contrasts (K = 90; Fig. 1*C*, bottom). As a consequence, the two bands displayed opposite spectral modulation compared with the intermediate contrast level (K = 30; Fig. 1*D*). To furtherly highlight this result, in the following we will adopt as reference contrast the value K = 30 (whose PSD is reported in Fig. 1*D*, middle) on which to compute the spectral modulation relative to the other levels of visual contrasts (see Materials and Methods).

### Broad and narrow band are modulated by complementary contrast ranges over different temporal windows

To furtherly investigate the differences between the processing of visual information of these two γ bands, we quantified the response over time of the narrow band on one hand, and the high ([65–95] Hz) and low ([20–45] Hz) broad band on the other (Fig. 2*A*).

First, we tested whether the sensitivity was the same over the whole range of visual contrasts explored when considering these γ band responses over the whole intercontrast-reversal interval (i.e., 500 ms). We found that NB at very low contrast (K = 0) had a significantly higher power than at intermediate contrast (K = 30; K = 0, 70 recordings across 12 animals: [−33.62 ± 0.52] dB/Hz vs K = 30, 79 recordings across 12 animals: [−36.33 ± 0.49] dB/Hz, median ± SEM, Kruskal–Wallis one-way ANOVA (K–W ANOVA1): *p *≪* *0.01, *post hoc* Dunn’s test, *p *≪* *0.01; Fig. 2*B*, top), while a small but not significant increase in PSD was found between contrasts from K = 30 to K = 90 (K = 30, 79 recordings across 12 animals: [−36.33 ± 0.49] dB/Hz vs K = 90, 32 recordings across 7 animals: [−35.78 ± 0.73] dB/Hz, *post hoc* Dunn’s test, *p *>* *0.5; Fig. 2*B*, top). This indicates that NB is not steadily decreasing for increasing contrast, but it is decreasing when contrast goes from low to intermediate level.

Conversely, low BB power was uniform for levels of contrast K ≤ 30 (K–W ANOVA1: *p *=* *0.02, *post hoc* Dunn’s test, *p *>* *0.5), while it increased significantly at high contrast (K ≥ 30; K = 90, 32 recordings across 7 animals: [−28.53 ± 0.74] dB/Hz vs K = 30, 79 recordings across 12 animals: [−31.26 ± 0.49] dB/Hz, *post hoc* Dunn’s test, *p *=* *0.04; Fig. 2*B*, middle). High range of BB ([65–95] Hz) was finally not significantly modulated by contrast when we considered the whole intercontrast-reversal interval (K–W ANOVA1: *p *=* *0.46; Fig. 2*B*, bottom). This suggests that NB and BB (at least its lower frequency component) not only display opposite correlations with contrast, but they are sensitive to complementary ranges of visual contrast levels.

We repeated this analysis separately for the intervals [0–200] and [200–500] ms following contrast reversal to discriminate between an early response because of the abrupt contrast reversal and a late one because of the presentation of a static visual contrast instead. NB PSD modulation was significant for both time windows (Fig. 2*C*, left, 350 recordings across 12 animals, K–W ANOVA1: *p *≪* *0.01; Fig. 2*C*, right, K–W ANOVA1: *p *≪* *0.01) although differences were broader in the late response ([0–200] ms K = 0: [−34.11 ± 0.62] dB/Hz, K = 30: [−36.77 ± 0.43] dB/Hz; [200–500] ms K = 0: [−33.20 ± 0.53] dB/Hz, K = 30: [−36.11 ± 0.49] dB/Hz).

Low BB early response was significantly modulated by contrast level (350 recordings across 12 animals, K–W ANOVA1: *p *=* *0.02; Fig. 2*D*, left), with the highest contrast (K = 90) displaying significantly higher power than at intermediate contrast level (K = 90: [−26.90 ± 0.80] dB/Hz vs K = 30: [−30.84 ± 0.48] dB/Hz, *post hoc* Dunn’s test, *p *=* *0.03; Fig. 2*D*, left). Low BB late response, instead, was not significantly modulated by contrast (K–W ANOVA1 *p *=* *0.06; Fig. 2*D*, right). Coherently, high BB was significantly modulated by contrast reversal only in the early response (Fig. 2*E*, left, 350 recordings across 12 animals, for the early response, K–W ANOVA1: *p *=* *0.04. K = 90: [−35.68 ± 0.71] dB/Hz vs K = 30: [−38.59 ± 0.46] dB/Hz, *post hoc* Dunn’s test, *p *=* *0.04. Fig. 2*E*, right, for the late response, K–W ANOVA1: *p *=* *0.30).

Overall, these results show that: (1) NB is only modulated by the low contrast range and is primarily modulated in the late response (i.e., by the presentation of a static visual contrast); (2) conversely, BB is only modulated by the high visual contrast range in the early phase of the response (i.e., in response to the abrupt reversal of the contrast grating).

### Broad and narrow band processing of visual information

The complementary sensitivity to visual contrast of narrow and broad band was furtherly investigated by estimating the information carried about the visual contrast level by the power of these two γ bands (for details, see Magri et al., 2009 and Materials and Methods). As high and low broad band have displayed a similar behavior, for this analysis (and in the following ones as well) they will be considered as one BB (as in Saleem et al., 2017).

Considering the whole intercontrast-reversal interval, NB carried significant information (*p *<* *0.05, bootstrap test; Materials and Methods) over the whole range of contrasts and for the low contrast range (K ≤ 30), but did not carry significant information about contrasts K > 30 (Fig. 3*A*). Interestingly, NB did not carry significant information at all when considering only modulations occurring in the first 200 ms after contrast reversal (Fig. 3*B*) proving that significant NB modulations occur only at a later stage. Indeed, information carried by NB in the late stage (from 200 to 500 ms after contrast reversal) about the visual contrast level was significant for all ranges considered, although the information about low level of contrast was higher (0.11 bits for K < 30 vs 0.08 bits for K > 30; Fig. 3*C*). This therefore proves that NB can be considered an information channel primarily dedicated to convey information about low levels of static visual contrast.

BB carried instead significant information only about contrasts K > 30 (Fig. 3*D*) when considering the whole intercontrast-reversal interval, suggesting that BB is instead an information channel dedicated primarily to convey information about high levels of contrast, complementarily to NB. The distribution of information over time of BB modulation was furthermore exactly complementary to NB, with significant information in the early response for all ranges (0.07 bits for K < 30 vs 0.11 bits for K > 30; Fig. 3*E*) and no significant information carried by late modulations (from 200 to 500 ms after contrast reversal; Fig. 3*F*).

Coherently with this complementarity, spectral modulations of the two bands displayed a synergistic contribution in the encoding of visual contrasts (0.29 bits over the whole stimulation window and contrast range), such in a way that, when considering the modulations of both bands, the large window carries significant information about all contrast ranges (Fig. 3*G*). Moreover, both early and late responses carry significant information (Fig. 3*H*,*I*). This result depends critically on the complementarity of the sensitivity ranges: if contrast-induced modulations in NB and BB were the opposite, with NB decreasing over the whole range and BB increasing over the whole range, the two bands would carry highly redundant information.

Altogether these results show NB and BB to be complementary information channels, with the former encoding increase in contrast from K = 0 to K = 30 by decreasing power >200 ms after contrast reversal, and the latter encoding increase in contrast from K = 30 to K = 90 by increasing power right after contrast reversal.

### Narrow and broad band γ dynamics at contrast reversal

As we observed a clear temporal structure of the two γ bands, we focused on the temporal evolution of NB and BB modulation (see Materials and Methods). As expected, narrow band modulation at K = 0 showed a sustained activity throughout the whole stimulation with no specific response associated with contrast reversal (Fig. 4*B*). At null visual contrast level, indeed, the contrast reversal does not produce any change in the visual stimulus. For K = 10, NB power instead decreased after the grating contrast reversal (minimum NB modulation: [−4.5 ± 2.3] % reached at [107 ± 5] ms; Fig. 4*C*). This decrease in NB power lasted for ∼200 ms (K = 10 offset: [198 ± 10] ms).

Broad band γ power expressed, instead, a rapid transient increase immediately after high contrast reversal [maximum BB modulation at K = 50 [17 ± 4] % reached at [73 ± 8] ms (Fig. 4*D*); maximum BB modulation at K = 90 [46 ± 9] % reached at [76 ± 9] ms (Fig. 4*E*)]. After this transient enhancement, BB power returned to the precontrast reversal baseline level within 200 ms (as previously observed for the NB; K = 50 offset: [177 ± 2] ms; K = 90 offset: [209 ± 1] ms).

Of note, NB and BB displayed different temporal dynamics relative to contrast reversal, with the former having a much longer delay (NB onset: [42 ± 5] ms for K = 10; BB onset: [4 ± 4] ms, for K = 50 and K = 90; Wilcoxon rank-sum test *p *<* *0.05).

### Spiking neuronal network captures distinct functional γ bands

To model the modulation with contrast of these functional γ bands in V1, we took into account the presence of a NB oscillation in rodent thalamic activity (Saleem et al., 2017; McAfee et al., 2018; see Materials and Methods), by incorporating it in the input of a spiking neuronal network for which it was shown the γ BB to be modulated by the input intensity (Mazzoni et al., 2008). Briefly, the simulated input was given by the superimposition of two components: a sustained component with an average intensity depending on contrast [S(K)], and an oscillatory component with a fixed frequency of ∼60 Hz and a contrast-dependent amplitude [A(K); Fig. 5*A*, left; Eq. 9].

The oscillatory component of the thalamic input A(K) of the model generated a NB peak in the simulated V1 LFP spectrum **(**Fig. 5*B*, left) with a strong correlation coefficient between the cortical NB peak power and the amplitude A of the thalamic NB (Pearson correlation, *R* = 0.94, *p *≪* *0.001). Note that V1 NB peak frequency is exactly the same as A dominant frequency (Fig. 5*B*, middle). Moreover, input and output oscillations were tightly phase-locked in the NB but not in the BB range (Fig. 5*B*, right). A two-way ANOVA analysis of the circular variance for factors (frequency band, amplitude of thalamic input) found a significant interaction (*p *=* *0.003) between the two factors. The NB band was significantly modulated by the amplitude of the periodic thalamic component A in the LFP NB (K–W test *p *≪* *0.001) but not the LFP BB (K–W test *p *=* *0.8). As the circular variance in the NB diminished as the thalamic periodic input increased in amplitude, the NB circular variance was consequently found to be significantly (*p *=* *0.002) less than the BB circular variance for A > 30, hence indicating a tighter phase locking. This shows that in our model the thalamic periodic input (modulated by the parameter A) generates a phase locked periodic response in V1 with the same peak frequency.

The sustained component of thalamic input S(K) modulated instead to the onset and the strengthening of cortically generated γ oscillations, as previously seen in this kind of model (Mazzoni et al., 2008), resulting in an increase of power in frequencies involving both BB and NB (linear correlation coefficient between BB power of the simulated LFPs and the sustained component of the thalamic input equal to 0.98, *p *=* *0.004; Fig. 5*C*, left). Interestingly, phase-locking between V1 NB ad BB and the associated components of the input decreased when modulating the sustained thalamic input strength (see the increase of circular variance in Fig. 5*C*, right), showing an opposite trend compared with the previous case in which the thalamic NB was modulated.

This shows that in our model, the periodic component of the thalamic input generates cortical NB oscillations through entrainment of the cortical activity, while the sustained component of the thalamic input leads to the onset of cortically generated γ oscillations encompassing both BB and NB.

By varying A(K) while S(K) was fixed (see Table 2), the model reproduced quantitatively (modulation at K = 0 $X_{r}^{2}=0.25$; modulation at K = 10 $X_{r}^{2}=0.28$) the experimentally observed NB modulation for low levels of contrast (Fig. 5*D*). For the same set of network parameters (see Table 1), the model quantitatively (modulation at K = 50 $X_{r}^{2}=0.14$; modulation at K = 90 $X_{r}^{2}=0.23$) reproduced BB peak shape and BB power increase from reference to high contrast (Fig. 5*E*). This was instead achieved by varying S(K) while A(K) was held fixed (see Table 2). This suggests that the different modulation of these two γ bands can be accounted for by the two different neural mechanisms proposed by the model: (1) the sustained thalamic input S(K), determining the intensity of the broad band γ through the modulation of endogenous cortical γ; (2) the periodic thalamic input of amplitude A(K), determining the intensity of the cortical narrow band through neural entrainment.

### Model of complementary contribution of oscillatory and sustained thalamic input to LFP γ activity

We then explored the possibility of capturing in the model the whole range of contrast-dependent γ modulation by reflecting in the thalamic input the result of the complementary range of contrast sensitivity we found in the cortex for NB and BB. We defined then a complementary range of contrast sensitivity for the oscillatory input A(K) and sustained input S(K). We set the oscillatory input A(K) to zero in the range K ≥ 30, while in the low contrasts K < 30 range we selected, for each K, the value of A maximizing the overall similarity between simulated and experimental spectral modulations (see Materials and Methods). We obtained a monotonous negative trend (see Table 2) that could be described by a piecewise decreasing linear function (Fig. 6*A*; Eq. 10). When coupled with a fixed value of S(K), this type of tuning of the A(K) parameter led to a decrease of the narrow band with simulated contrast (K–W ANOVA *p *>* *0.9 for K ≥ 30, K–W ANOVA *p *=* *0.08 for K ≤ 20, K–W ANOVA *p *<* *0.01 for K ≤ 10; Fig. 6*C*, top) and negligible fluctuations in the broad band instead (K–W ANOVA *p *>* *0.9; Fig. 6*C*, bottom).

Conversely, we set the sustained input S(K) to have a fixed baseline value S(K) = 500 sp./s up to K = 30, while in the high contrast range K > 30, for each simulated K value, we selected the value of the sustained input S maximizing the overall similarity between the simulated and experimental spectral modulations. We obtained a monotonous positive trend that could be described by a linear fit (Fig. 6*B*; Eq. 11). As expected, cortical γ band increased with S(K) (linear correlation coefficient equal to 0.98, *p *<* *0.001). This finally led to an increase of both NB (K–W ANOVA *p *=* *0.06 for K ≤ 70 and K–W ANOVA *p *≪* *0.001 for K ≤ 90; Fig. 6*D*, top) and BB (K–W ANOVA *p *=* *0.09 for K ≤ 60 and K–W ANOVA *p *≪* *0.001 for K ≤ 90; Fig. 6*D*, bottom) for simulated contrast levels K increasing in the high contrast range. This small increase in NB in the high contrast range is in agreement with results in Figures 2*B*,*C*, left panel, and 5*C*, and it is a by-product of the increase of activity in whole γ range (see Discussion).

We expect A(K) and S(K) to be present at each time for any level of input in real conditions. To compare experimental and simulation results over the whole range of contrasts we then injected in the network, for each value of K, the thalamic input resulting from the superimposition of both A(K) and S(K) values as defined above. We found that the network reproduced the experimental modulations of both the NB and the BB over the whole set of contrasts ( $X_{r}^{2}=0.07$ and $X_{r}^{2}=0.12$; Fig. 6*E*, top and bottom, respectively). Similar results were achieved replacing the values of A(K) and S(K) optimized for each value of K with two piecewise linear functions ( $X_{r}^{2}=0.14$ and $X_{r}^{2}=0.23$; Fig. 6*E*, top and bottom, respectively). These results show (1) that the dynamics of γ bands in mouse V1 can be reproduced by a standard network architecture by a proper design of thalamic inputs; (2) that very good agreement with experimental data are achieved by modeling thalamic input as the sum of an oscillatory component, sensitive to low levels of contrast, and a sustained component, sensitive, instead, to high levels of contrast.

A broader exploration of input parameters revealed that in general NB is sensitive to the increase in the periodic input A(K) and, to a lesser extent, to the increase in the sustained input S(K) (two-way ANOVA showed *p *≪* *0.001 for parameter A, *p *≪* *0.001 for parameter S and *p *=* *0.3 for their interaction), while BB is only sensitive to the sustained input S(K) (two-way ANOVA showed *p *=* *0.33 for A, *p *≪* *0.001 for S and *p *=* *0.32 for their interaction).

### Time-dependent thalamic input model accounts for γ band temporal evolution

Modeling results shown in Figures 5, 6 reproduced the average response of the network over time. To reproduce the experimental time-frequency features of V1 LFPs we re-defined the simulated thalamic inputs to be also a function of time (see Eq. 13) by taking into account a reversal-driven modulation at contrast reversal. The function was defined in such a way to be zeroed after 200 ms (as observed experimentally in the LFP scalograms; Fig. 7, last column). We then re-determined A(K,t) and S(K,t) to optimally fit experimental data (see Materials and Methods). This allowed capturing the differences in early and late thalamic stimulation leading to the distinction of early and late cortical responses observed experimentally.

By introducing this temporal dynamic in the thalamic input, we were able to reproduce the experimental LFPs at contrast reversal, both the NB time invariance at K = 0 (Fig. 7*A*, middle, and $X_{r}^{2}=0.03$ in Fig. 7*A*, right), the NB power decrease at K = 10 (Fig. 7*B*, middle, and $X_{r}^{2}$ = 0.02 in Fig. 7*B*, right. $X_{r}^{2}$ = 0.01 for K = 20; data not shown) and the BB power increase for high contrast associated with abrupt contrast reversal (K = 50: Fig. 7*C*, middle, and $X_{r}^{2}$ = 0.07 in Fig. 7*C*, right, K = 90: Fig. 7*D*, middle, and $X_{r}^{2}$= 0.21 in Fig. 7*D*, right).

## Discussion

We found that, in V1 of awake mice, narrow and BB displayed an opposite modulation with contrast and were sensitive to complementary contrast ranges with complementary temporal dynamics. Such complementarity of the two γ bands is not completely described by a narrow band encoding luminance opposed to a broad band encoding contrast as previously thought (Welle and Contreras, 2017): both bands actually encode contrast, but in a complementary way. To shed light on the network dynamics underlying these experimental results, we developed a simple leaky integrate-and-fire spiking network model of mice V1, which reproduced quantitatively the complementary contrast-driven γ modulations of the two bands. The model suggests that cortical γ bands complementarity originates in the thalamus, as the sustained and periodic components of the thalamic input are already sensitive to two complementary contrast ranges.

### Contrast sensitivity: experimental results

This work focused on the mice V1 processing of visual contrast. The power of the NB was found to decrease with contrast in the low contrast range, reach a plateau for medium contrast, and be weakly modulated by high contrast (Fig. 2*B*). This result differs from the claim of monotonic decrease of NB observed in (Saleem et al., 2017). This discrepancy of the results might be then because of a lack of a dedicated analysis in their work more than to difference in the experimental set-up: Saleem et al. (2017; see their Fig. 2*C*) hardly shows a significant decrease in NB power for contrasts above 50 (error bars being SEM).

On the other hand, the BB was found to be sensitive to the variation of high levels of visual contrast. While in (Saleem et al., 2017) the role of BB and NB in providing contrast information seemed redundant, we show that their role is instead complementary. Information theory indeed revealed the two γ bands to encode visual contrast in two separated ranges (Fig. 3): the low contrast range (K < 30), in which only the NB is modulated, and the high contrast range (K > 30) in which, instead, only the BB is modulated. These results suggest that in rodents NB might have a critical role not only in the encoding of luminance, as previously thought, but also in the encoding of contrast, thanks to a complementary sensitivity to the one of BB.

The NB and the BB are not only modulated by complementary contrast ranges but also primarily modulated in two complementary temporal windows. At low contrasts, NB is indeed desynchronized in the early phase after stimulus presentation, but the prominent effect is a significant reduction of power in the late response compared with baseline (Figs. 3*C*, 4*C*). At high contrast, the increase in the BB is instead primarily modulated in the first 200 ms after stimulus presentation (Figs. 3*E*, 4*D*).

### Circuit dynamics leading to contrast sensitivity: predictions from the model

Our model provides a complete candidate description of the way different levels of contrast affect LGN activity and of how this leads to NB and BB modulation in V1.

First, the mechanisms of BB γ modulation in rodents V1 is the same that in primates V1, i.e., the increase in the intensity of the thalamic input (i.e., the increase of excitatory external drive to the cortical circuitry) because of higher contrast enhances endogenous γ oscillations (Henrie and Shapley, 2005; Mazzoni et al., 2008). This makes BB power proportional to the average value of S(K) (Figs. 5, 6). However, the increase in thalamic input intensity in rodents is not linear but is negligible for low contrasts. Hence V1 BB in rodents is modulated only by high contrast.

Second, there are two different mechanisms originating NB, depending on the level of contrast. At low contrasts, the dominant thalamic input is the periodic one (Fig. 6), which entrains V1 NB oscillation (Fig. 5*B*). Hence, cortical NB power at low contrast decreases with contrast as it reflects the contrast-driven modulation in power of thalamic NB oscillations (Fig. 6, left column). At high contrast, the dominant thalamic input is instead the sustained one (Fig. 6), which generates cortical γ oscillations, encompassing both NB and BB (Fig. 5*C*). Note that we do not rule out the possibility that LGN NB neurons have the capacity of encoding contrast variations in the high contrast range by furtherly desynchronizing. However, the associated decrease in cortical NB power becomes for high contrasts negligible compared with the increase because of local resonances triggered by the sustained thalamic input. Feed-forward-dependent responses are then overridden by recurrent connectivity-dependent components and cortical NB power at high contrast increases with contrast. Consequently, in the model, we divided the contrast range into two domains: a low one modulating only the periodic thalamic component and a high one modulating only the sustained thalamic component (Fig. 5). In other words, cortical NB power at high contrast increases with contrast as it reflects the increased endogenous γ activity because of the increased sustained thalamic drive.

These two ranges correspond also to two different operational modes of V1. In the low contrast range V1 passively mirrors the NB component in the LGN input. In the high contrast range the sustained input drives V1 to become an active intrinsic resonator in the BB (Brunel and Hakim, 1999).

We provide hence a prediction and an explanation for the γ band activity in V1 and thalamus given any contrast, providing several testable predictions, such as the complementary piecewise linear modulation of intensity and oscillations in the thalamus as a function of contrast.

Our model also reproduces the temporal evolution of spectral response (Fig. 7), under the assumption that both periodic and sustained thalamic inputs can be decomposed into a static component not varying in time and a component specifically associated to the moment of contrast reversal (see Eqs. 12, 14, respectively). It is tempting to speculate that the former is driven by spatial contrast and the latter by temporal contrast. The reversal-locked component might indeed stem from a sudden surge of the thalamus activity right after contrast reversal (maybe because of the peak in temporal contrast; Barbieri et al., 2014), which leads to an increase of the baseline input [i.e., S(K) in Eq. 14] and a temporary disruption of local thalamic NB γ oscillation [i.e., A(K) in Eq. 12]. This is compatible with the observed increase of multiunit activity in the early window after contrast reversal as a function of visual contrast level (data not shown).

Our experimental data on the processing of visual contrast are overall coherent with what previously described; however, our analysis adds novel and important insights into the dynamics and the functional role of the NB in encoding this feature.

### Luminance sensitivity

One of the main limitations of this study is that we did not explicitly tackle the luminance sensitivity of the two bands as we performed experiments with fixed luminance. However, we observed that stimulation onset (i.e., turning on the screen which is associated to an increase of luminance) was associated with an increase in both NB and BB (Fig. 1*B*). Previous studies (Saleem et al., 2017; Storchi et al., 2017) found that LGN NB was modulated by light intensity, with a higher luminance being associated with a narrow band with larger power and higher peak frequency (Saleem et al., 2017). Our model predicts that thalamic narrow oscillations entrain the cortical NB with the same peak frequency (Fig. 5*B*, middle). Hence, we would expect the light-induced increase in LGN γ activity observed by Saleem et al. (2017) to drive proportional increases in the cortical NB. As our work highlighted how the narrow band is modulated by both luminance and contrast, future experiments should take into account both these visual features to thoroughly describe their interplay.

### Laminar γ in rodents

γ Activity is known to spread non homogeneously across layers both when it is spontaneous (Welle and Contreras, 2016; Senzai et al., 2019) and when it is stimuli induced (Xing et al., 2012b; Bastos et al., 2014; van Kerkoerle et al., 2014; Saleem et al., 2017). We decided here to focus on Layer IV in both analysis and modeling (see Materials and Methods), as this layer receives direct thalamic inputs and displays early and strong narrow band γ oscillations when presented with visual contrast stimuli (Saleem et al., 2017). However, modeling studies suggest that contrast-dependent interlayer coupling might play a role in the shift between NB and BB at high contrasts. In particular, the temporal decorrelation in population activity at high contrasts leads to γ peak broadening (Battaglia and Hansel, 2011). Future works will then investigate interlayer properties of propagation of the two bands, analyzing the activity of different recording sites in laminar electrodes and exploiting the knowledge of interlaminar interactions of advanced models of mice V1 (see below, Models of mouse visual cortex).

### Models of mouse visual cortex

Recent models of mouse visual cortex (Billeh et al., 2020; but see also Troyer et al., 1998; Arkhipov et al., 2018), succeeded in reproducing firing rate distribution across cortical layers in response to arbitrary visual inputs with a multilayer network composed by multicompartment generalized LIF neurons. In these models LGN inputs are modeled as a series of spatiotemporal filters of the presented image and lack the γ band activity observed in LGN (Saleem et al., 2017; McAfee et al., 2018). γ modulation because of visual stimuli is indeed present in the model only with a sensitivity similar to the experimentally observed broad band, coherently with our hypothesis that the anti-correlation between V1 narrow band power and contrast might originate from periodicity in the LGN input. Combining our proposed model of LGN inputs with multilayer models as (Billeh et al., 2020) could unravel the specific interlayer dynamics of the narrow band.

Hippocampal models (Keeley et al., 2017) were able to reproduce the shift between slow and fast γ oscillations observed in the area (Colgin et al., 2009). These two rhythms (roughly corresponding to low and high BB in our work) are both generated within the hippocampus because of differences in inhibitory synaptic times (Keeley et al., 2017) rather than by entrainment to external inputs.

A recent interesting modeling study reproduced the effects of LGN input modulation on V1 γ responses to visual stimuli (Zachariou et al., 2021) with a single-layer network of conductance-based spiking neurons. However, the model aimed at reproducing the saturation of γ power at mid-level of visual contrast that has been observed in human and nonhuman primates (Hadjipapas et al., 2015) and was not found in our data nor similar murine data (Saleem et al., 2017; McAfee et al., 2018).

### NB and BB in primates

Encoding of contrast in the V1 γ band is known to be present in primates (Henrie and Shapley, 2005). A comparison of the role of narrow and BB between rodents and primates is however hampered by the fact that nomenclature is different in the two cases. In primates’ studies, NB and BB are defined as low versus high γ ranges: [30–80] versus [80–200] Hz (Ray and Maunsell, 2010), [20–60] versus [70–150] Hz (Bartoli et al., 2019), or even as overlapping [30–80] versus [30–200] Hz (Hermes et al., 2019).

Nevertheless, many studies have observed that in primates a sudden increase in visual contrast leads to a transient broad band activation followed by a sustained narrow band activity (Swettenham et al., 2009; Ray and Maunsell, 2010; Xing et al., 2012a,b; Murty et al., 2018; Shirhatti and Ray, 2018; Peter et al., 2019). Unlike mice, however, the power and the peak frequency of the sustained γ narrow band were found to be positively modulated by visual contrasts and invariant to luminance variation (Peter et al., 2019).

The V1 spiking neurons network we adopted here is not significantly different from previously proposed ones proved to reproduce contrast modulations in primates V1 (Mazzoni et al., 2008, 2010, 2011), except for the different thalamic inputs. This implies that we would expect to observe NB as defined in this manuscript also in primates if NB oscillations were present in primates’ LGN. However, while the sustained narrow γ oscillation has been reported in pre cortical structures such as LGN or even the retina for rodents and cats (Neuenschwander and Singer, 1996; Storchi et al., 2017), simultaneous recording from primate V1 and LGN found no narrow band in V1 and no γ oscillation in LGN (Bastos et al., 2014).

### Perspectives

Mice are becoming the favorite animal model to study the circuit changes involved in several neurologic disorders. This is because of the availability of sensitive imaging techniques and opto-genetic and chemo-genetic approaches identifying that allow the cellular underpinnings of the disease (Götz et al., 2018; Fagiolini et al., 2020). Mice are also becoming a standard model for the visual cortex (Carandini and Churchland, 2013; Haider et al., 2013; Saleem et al., 2017). In this context, the monitoring of visual responses represents a promising biomarker for preclinical and clinical studies on neurodevelopmental disorders (Lupori et al., 2019). Thus, an effective in silico model reproducing cardinal functions of the mouse visual cortex could lay the ground for a better understanding of the pathogenetic mechanisms underlying functional impairment in neurologic diseases. In future works, we aim at investigating disorders involving the visual cortex with an interplay of experimental and modeling studies starting from the results presented here.

## Synthesis

Reviewing Editor: William Stacey, University of Michigan Decisions are customarily a result of the Reviewing Editor and the peer reviewers coming together and discussing their recommendations until a consensus is reached. When revisions are invited, a fact-based synthesis statement explaining their decision and outlining what is needed to prepare a revision will be listed below.

We apologize for the extended delay. This delay is not in line with eNeuro guidelines, but resulted because one of the reviewers never responded and we had to acquire a new reviewer. I deeply apologize. However, in our synthesis we agreed that the paper was very interesting and just needed some minor clarifications.

Could you add some more comments on the mechanisms? The NB gamma is essentially a feed-forward process, while the BB is a recurrently generated spectrum component. Why would the NB component lose the capacity to encode large contrast fluctuations? Are feed-forward dependent responses overridden by recurrent connectivity dependent components of responses? If yes, there is an interesting switch between roles of local V1 connectivity, from mere filter of inputs at low contrast to active intrinsic resonator at high contrast that could be commented on more explicitly.

The discussion mentions multi-layered circuits as a perspective. In the past it has been proposed that multi-layer structure may have an impact in shaping oscillatory responses. Notably at weak effective coupling between layers, L4 would act as a pure gamma generator giving rise to NB gamma. However at stronger inter-layer coupling, the NB component would leak its power toward other frequencies and be gradually replaced by a BB gamma component (associated to chaotic gamma bursting arising). This switch from regular NB to chaotic BB may be an alternative explanation for the observed findings, and could be possible if the contrast of the stimulus beyond increasing the drive to each layers also effectively gates interlayer coupling. What do the authors think of this alternative mechanism (that could be mentioned in Discussion?)

A few minor comments about information theory quantities:

- It would be nice to explain with more precision how the binning for power levels was performed in estimating mutual information with contrast. Indeed when the response is not linear a uniform binning may hide the existence of a coding but with a different dynamic range. Specifically, if using large bins is sufficient to capture big variations of power (e.g. at low contrast for NB and at high contrast for BB), it may not be enough to capture small variations of power (that could still occur e.g. for NB at high contrast and for BB at low contrast). The use of a non uniform binning may reveal that there is maybe still a coding of contrast by BB and NB even in their non-preferred K range, but that variations are smaller and thus susceptible to be lost in noise. This would be a slightly weaker claim than lack of coding. If willing to keep the stronger claim of total lack of coding then additional care should be taken using rather than uniform “plug-in” bins adapted size non-uniform bins or kernel estimators with adaptive kernel size.

There is one potential minor disagreement in mutual information. Strictly speaking the synergistic contribution estimated as *S**y**n*(*K*; *N**B**K* , *B**B**K* ) = *I*(*K*; *N**B**K* , *B**B**K* ) − [*I*(*K*; *N**B**K*) + *I*(*K*; *B**B**K*)] is not completely correct because it does not take into account that the mutual information terms *I*(*K*; *N**B**K*). *I*(*K*; *B**B**K*) do not consist exclusively of “unique information” and could also have redundant “shared information”. The redundant shared part is then counted and subtracted twice. There is no simple way to estimate this shared part however it is bounded by Ired = min[*I*(*K*; *N**B**K*), *I*(*K*; *B**B**K*)]. So a more correct estimate for Synergy would be Synergy_corrected = Synergy + Ired. This may slightly change the results (potentially boosting even synergy estimation, as the correction is positive? Or removing negative synergy effects, not mentioned by the authors but that could be present). If the correction is not applied, the potential misestimation artefact should at least be mentioned. Could you comment on this concern?

The following comments should be addressed:

Additional experimental details. “Each of the twelve animals underwent two recording sessions on two different days.” Is this 2 recording sessions on each of two days or in total across two days? During each session, how many 30 second trials were presented at each contrast level? Were all contrast levels tested within a session, and if so, were they randomized? The specific contrast levels tested appear missing from the methods; we assume from Figure 2 that 6 levels were tested in total.

It was hard to find the source of the total number of recordings (e.g. Figure 2 C, left, 350 recordings across 12 animals). Our assumption was that this number would reflect 4 recording sessions per animal x 6 contrast levels (5 for 5 of the animals) x 12 animals, but that yields 268 recordings.

Fig 1b shows the scale in dB but dB normalization was not described in the methods.

Extracting broadband and narrowband gamma. Authors may want to consider modeling oscillatory and broadband gamma activities through an approach such as fooof (Donoghue et al., 2020, Nature Neuroscience); the current band limited approach matches previous studies but may lose some information.

Fig 1B

"narrow band (middle, black) and broad band (black). “ Should this be broad band (gray)?

Code availability

Neither reviewer was able to access the code. It is necessary for code to be available, and we frequently find that other researchers have difficulty implementing code. Please upload to an anonymized hub (e.g. github) so the code can be tested. Alternatively you could remove the double blind.

## Author Response

Dear Editors,

Thanks for this precious opportunity to improve our manuscript to reach the quality necessary for the publication on eNeuro. Below you find in red our point-by-point replies to the comments.

Thanks again, kindest regards,

The corresponding author

Editorial comments

1) If you would like to include a visual abstract with your revision, you can refer to instructions found here: https://www.eneuro.org/content/preparing-manuscript#visual

We are grateful for this opportunity. We prepared this graphical abstract:

2) In an effort to improve rigor, transparency, and reproducibility, eNeuro strongly encourages the use of estimation statistics. Please refer to the editorial (https://doi.org/10.1523/ENEURO.0259-19.2019) and commentary (https://doi.org/10.1523/ENEURO.0205-19.2019) for the why and how.

If the use of estimation statistics applies to your research, you may consider including it for the revision. We will regularly highlight these papers to the community to show how helpful estimation statistics can be, increasing the exposure of your work.2

We thank the editor for pointing this issue out. We are of course sympathetic with the mission of eNeuro of improving inference statistics by endowing null-hypothesis statistical testing with quantitative estimation ofeffect sizes and their uncertainties. The only section of our work for which estimation statistics might apply is Broad and narrow band are modulated by complementary contrast ranges over different temporal windows. Actually, we have already reported the data ranges for each comparison in the section. Following the editor’s suggestion, now we also modified our Figure 2 by using the freely available estimation graphic software DABEST (Ho et al., 2019) that is now mentioned in Materials and Methods -> Network model ->

Statistical analysis:

Statistical analysis [...]. As for the results presented in Figure 2 we adopted the estimation graphics package presented in (Ho et al., 2019).

We modified the caption of Figure 2 accordingly:

Figure 2. Narrow and broad band gamma contrast-driven modulation in layer IV of mouse V1.

(B) PSD of NB (left), low BB (middle), high BB (right) as a function of visual contrast pooled across all animals and recordings over the whole [0 500] ms stimulation interval. On upper boxes, the horizontal red line indicates the median, bottom and top edge indicate the 25th and 75th percentiles. The whiskers extend to the most extreme data points not considered outliers (which are represented by red pluses). Asterisks indicate significant post-hoc pairwise difference (Kruskal-Wallis followed by Dunn’s test p<0.05). ‘n.s.’ indicates non-significant statistical difference across contrasts. Individual experimental data points are indicated as gray circles. Lower boxes display the mean difference in power spectral density, ΔPSD, between a reference contrast (K=0 for NB and K=30 for the BB) and the other contrast levels. Black whiskers indicate the 95% confidence interval obtained though non-parametric bias-corrected bootstrap resampling (Ho et al., 2019).

(C-E) Same as (B) for narrow band (C), low broad band (D) and high broad band (E) dividing the response interval in two windows: [0 200] ms (left) and [200 500] ms (right).3

Reviewers comments

---------------------------------------------

Synthesis of Reviews:

Computational Neuroscience Model Code Accessibility Comments for Author (Required):

Neither reviewer was able to access the code. It is necessary for code to be available, and we frequently find that other researchers have difficulty implementing code. Please upload to an anonymized hub (e.g. github) so the code can be tested. Alternatively you could remove the double blind.

We thank the reviewers for this comment. The code was and is still attached as extended data. Now we have also uploaded it to Anonymous Github (/r/Mice-V1-Narrow-Broad-Gamma-Band/.)

Synthesis Statement for Author (Required):

Could you add some more comments on the mechanisms? The NB gamma is essentially a feed-forward process, while the BB is a recurrently generated spectrum component.

Why would the NB component lose the capacity to encode large contrast fluctuations? Are feed-forward dependent responses overridden by recurrent connectivity dependent components of responses?

We thank the reviewer for this comment. The switch between V1 NB entrainment and BB resonance is central to our manuscript and we agree that more detailed explanations upon the mechanism generating such transition are beneficial for the manuscript.

Narrow band gamma oscillations in mice visual cortex are indeed a feed forward process as they are transmitted by the rhythmic firing of subcortical neuronal ensembles (such as the LGN and, possibly, the retinal ganglion cells in the retina). The narrow band component of the LGN input to V1 was already known

to desynchronize with increasing levels of visual spatial contrast (Saleem et al., 2017). From the experimental analysis of our data, we also found this desynchronization to be observable in the narrow band of the cortex from low (K=0) to mid (K=30) visual contrast (see Figure 1 and Figure 2). On the other hand, we found that furtherly increasing the contrast level resulted in a spectral modulation of a broader (hence the name BB) range of frequencies, also encompassing the NB gamma frequency domain (please see again Figure 1 and Figure 2). We do not rule out the possibility that LGN NB neurons have the capacity of encoding contrast variations in the high contrast range by further desynchronizing. However, the associated decrease in cortical NB power becomes for high contrasts negligible compared to the increase due to local resonances triggered by the sustained thalamic input. As the reviewer correctly says, at high contrast “feed-forward dependent responses (are) overridden by recurrent connectivity dependent components of responses”. Hence, in the model we divided the contrast range into two domains: a low one modulating only the periodic thalamic component, and a high one modulating only the sustained thalamic component (please see Figure 5).

We added the following sentence in the Discussion section -> Circuit dynamics leading to contrast sensitivity: predictions from the model

"Note that we do not rule out the possibility that LGN NB neurons have the capacity of encoding contrast variations in the high contrast range by furtherly desynchronizing. However, the associated decrease in cortical NB power becomes for high contrasts negligible compared to the increase due to local resonances triggered by the sustained thalamic input. Feed-forward dependent responses are then overridden by recurrent connectivity dependent components and cortical NB power at high contrast increases with contrast. Consequently, in the model we divided the contrast range into two domains: a low one modulating 4

only the periodic thalamic component, and a high one modulating only the sustained thalamic component (Figure 5).”

If yes, there is an interesting switch between roles of local V1 connectivity, from mere filter of inputs at low contrast to active intrinsic resonator at high contrast that could be commented on more explicitly.

We agree with the reviewer on the presence of this switch of V1 role that is due to the proprieties of the

thalamic input, dominated by the periodic component at low contrasts and by the sustained component at high contrast.

The former component is responsible for transmitting to the V1 network a series of excitatory post synaptic currents oscillating in the NB gamma range, entraining the dynamics of the network. In this contrast range

the V1 network can then be thought of as a mere passive component simply mirroring the proprieties of its oscillatory input.

The latter component, instead, triggers local resonances by injecting the cortical network with increasing levels of energy (Brunel and Hakim, 1999). In this contrast range, therefore, the V1 network is no longer a simple mirror of its input, but, due to the network proprieties involving the balance between excitatory and inhibitory neurons, becomes an active resonator.

We briefly report this issue in the Discussion section -> Circuit dynamics leading to contrast sensitivity: predictions from the model as follows:

"These two ranges correspond also to two different operational modes of V1. In the low contrast range V1 passively mirrors the NB component in the LGN input. In the high contrast range the sustained input drives V1 to become an active intrinsic resonator in the BB (Brunel and Hakim, 1999).”

The discussion mentions multi-layered circuits as a perspective. In the past it has been proposed that multi-layer structure may have an impact in shaping oscillatory responses. Notably at weak effective coupling between layers, L4 would act as a pure gamma generator giving rise to NB gamma. However at stronger inter-layer coupling, the NB component would leak its power toward other frequencies and be gradually replaced by a BB gamma component (associated to chaotic gamma bursting arising). This switch from

regular NB to chaotic BB may be an alternative explanation for the observed findings, and could be possible if the contrast of the stimulus beyond increasing the drive to each layers also effectively gates interlayer coupling. What do the authors think of this alternative mechanism (that could be mentioned in Discussion?)

We thank the reviewer for this interesting comment. We are aware of the study suggesting that this transition between regular NB to chaotic BB occurs in V1 as visual contrast increases (Battaglia and Hansel, 2011) . This phenomenon might well occur in rodents V1. However, results supporting the subcortical origin of cortical NB in mice are robust (Saleem et al., 2017; Storchi et al., 2017). We think therefore that the onset of chaotic gamma bursting might be relevant primarily to explain why the BB is actually so broad. This is reported in the Discussion -> Laminar gamma in rodents as follows:

"Gamma activity is known to spread non homogeneously across layers both when it is spontaneous (Welle and Contreras, 2016; Senzai et al., 2019) and when it is stimuli-induced (Xing et al., 2012b; Bastos et al., 2014; van Kerkoerle et al., 2014; Saleem et al., 2017). We decided here to focus on layer IV in both analysis and modelling (see Methods), as this layer receives direct thalamic inputs and displays early and strong narrow band gamma oscillations when presented with visual contrast stimuli (Saleem et al., 2017).

However, modelling studies suggest that contrast dependent inter-layer coupling might play a role in the shift between NB and BB at high contrasts. In particular, the temporal decorrelation in population activity at high contrasts leads to gamma peak broadening (Battaglia and Hansel, 2011). Future works will then investigate inter-layer properties of propagation of the two bands, analyzing the activity of different 5

recording sites in laminar electrodes and exploiting the knowledge of inter-laminar interactions of advanced models of mice V1 (see next subsection)”.

A few minor comments about information theory quantities:

- It would be nice to explain with more precision how the binning for power levels was performed in estimating mutual information with contrast. Indeed when the response is not linear a uniform binning may hide the existence of a coding but with a different dynamic range. Specifically, if using large bins is sufficient to capture big variations of power (e.g. at low contrast for NB and at high contrast for BB), it may not be enough to capture small variations of power (that could still occur e.g. for NB at high contrast and for BB at low contrast). The use of a non uniform binning may reveal that there is maybe still a coding of contrast by BB and NB even in their non-preferred K range, but that variations are smaller and thus susceptible to be lost in noise. This would be a slightly weaker claim than lack of coding. If willing to keep the stronger claim of total lack of coding then additional care should be taken using rather than uniform “plug-in” bins adapted size non-uniform bins or kernel estimators with adaptive kernel size.

We thank the reviewer for raising this point and we apologize for not describing more clearly the discretization procedure applied in our work. In Materials and Methods ->Neurophysiological data analysis -> Spectral information analysis we replaced "The probabilities, both marginal and conditional, were computed by discretizing the power modulations into 10 equipopulated bins.” with"The probabilities, both marginal and conditional, were computed by discretizing the power modulations into 10 equipopulated bins. Specifically, the discretization boundaries were chosen so that each bin contains the same number of elements. This discretization choice, in place of an “equal-width” binning procedure, ensures robustness against outlier and maximizes the response entropy (Timme and Lapish, 2018).”

We therefore now hope to have more clearly stated that we did not adopt a uniform binning procedure, in the sense of equi-spaced. Furthermore, we decided to use a number of bins (i.e., 10) that seemed sufficient to capture small non-linear variations in the probability distribution of the response variables conditioned to the stimuli, given the set of trials that we had (>30/stimulus). For full disclosure, we computed the mutual information as a function of different number of bins for our multi-dimensional case (i.e., I(K;NB,BB) of Figure 3 G-I). As for the manuscript, we adopted equi-populated discretization of power distribution and the bias was accounted for by applying the Panzeri-Treves correction. 6

From the attached figure, it can be appreciated the mutual information is sensitive to low (n<4) and high (n>15) number of bins adopted. The choice adopted in the analysis of the main manuscript (n=10) lies instead within a domain where mutual information estimate reach a saturation plateau are less prone to be affected by this parameter. We therefor opt to keep this number of bins.

Following the suggestion of the reviewer we also evaluated the information with a binless procedure (Victor, 2002). The procedure systematically overestimated the information. As pointed out (Victor, 2002)

the binless procedure is associated to an higher variability in the estimates, that, for high dimensional codes, is compensated by a reduction in the bias. As here we are considering a one/two-dimensional code, we decided to adopt the more conservative non-uniform binned estimate.

There is one potential minor disagreement in mutual information. Strictly speaking the synergistic contribution estimated as *S**y**n*(*K*; *N**B**K* , *B**B**K* ) = *I*(*K*; *N**B**K* , *B**B**K* ) − [*I*(*K*; *N**B**K*) + *I*(*K*; *B**B**K*)] is not completely correct because it does not take into account that the mutual information terms *I*(*K*; *N**B**K*). *I*(*K*; *B**B**K*) do not consist exclusively of “unique information” and could also have redundant “shared information”. The redundant shared part is then counted and subtracted twice. There is no simple way to estimate this shared part however it is bounded by Ired = min[*I*(*K*; *N**B**K*), *I*(*K*; *B**B**K*)]. So a more correct estimate for Synergy would be Synergy_corrected = Synergy + Ired. This may slightly change the results (potentially boosting even synergy estimation, as the correction is positive? Or removing negative synergy effects, not mentioned by the authors but that could be present). If the correction is not applied, the potential misestimation artefact should at least be mentioned. Could you comment on this concern?

We thank the reviewer for raising this important concern. We agree with the reviewer that our formulation of synergy could be over conservative as it did not take into consideration the redundant information shared by the individual response variables. For this reason, we considered to be most profitable for the robustness of our results to apply the correction suggested in the comment. We therefore estimate now the redundancy as proposed by (Williams and Beer, 2010). We changed the main text accordingly.

Specifically:

-) we modified Materials and Methods ->Neurophysiological data analysis -> Spectral information analysis as such:

Finally, the synergistic contribution in the mutual information when jointly considering both narrow and broadband gamma modulation was computed as:

*S**y**n*(*K*; *N**B**K*, *B**B**K*) = *I*(*K*; *N**B**K*, *B**B**K*) − [*I*(*K*; *N**B**K*) + *I*(*K*; *N**B**K*)] + *R**E**d*(*I*(*K*; *N**B**K*),*I*(*K*; *B**B**K*)) (3) where *I*(*K*; *N**B**K*, *B**B**K*) is the joint information carried by both narrow and broad band modulation, *I*(*K*; *N**B**K*) + *I*(*K*; *N**B**K*) is the sum of the information carried about visual contrast level by narrow and broad band gamma modulation respectively, and *R**E**d*(*I*(*K*; *N**B**K*),*I*(*K*; *N**B**K*)) is the amount of redundancy (i.e., the amount of information overlap existing between the two gamma bands power about the stimulus levels).

We have employed as a measure of redundancy the one proposed by (Williams and Beer, 2010), namely the minimum information: redundancy is expressed as the expected value of the minimum information that any response variable carries individually about each one of the stimuli.

*R**E**d*(*I*(*K*; *N**B**K*),*I*(*K*; *B**B**K*)) = ∑ *p*(*k*) ∗ min{*I*(*K* = *k*, *N**B**k*);*I*(*K* = *k*, *B**B**k*)} *k* (4)7-) We computed therefore the synergy with this new definition and changed the value reported in the manuscript accordingly.

Coherently with this complementarity, spectral modulations of the two bands displayed a synergistic contribution in the encoding of visual contrasts (0.29 bits over the whole stimulation window and contrast range), such in a way that, when considering the modulations of both bands, the large window carries significant information about all contrast ranges (Figure 3 G).

The corrected values of synergy did not affect qualitatively the results and left our conclusions unchanged.

The following comments should be addressed:

Additional experimental details. “Each of the twelve animals underwent two recording sessions on two different days.” Is this 2 recording sessions on each of two days or in total across two days? During each session, how many 30 second trials were presented at each contrast level? Were all contrast levels tested within a session, and if so, were they randomized? The specific contrast levels tested appear missing from the methods; we assume from Figure 2 that 6 levels were tested in total.

We thank the reviewer for pointing this out. We agreed with the suggestion of improving the clarity of the experimental procedure.

The Materials and Methods -> Experimental design -> Extracellular recordings in awake mice paragraph now reads:

Each of the twelve animals underwent two recording sessions on two different days (one per day). Each experimental session was made up of at least two visual contrast sweeps. Specifically, each sweep consisted in presenting the mice with five contrast levels [0 10 20 30 50] % (n=5 animals) or six contrast levels [0 10 20 30 50 90] % (n=7 animals) in increasing order. Each visual stimulus consisted of 1 Hz alternating gratings presented for 30 seconds at a single contrast level to the head-fixed animals (see Visual Stimuli). Overall, for each level of contrast below K=90 we have 2 recording sessions x ({greater than or equal to}2 sweeps) x 12 animals {greater than or equal to} 48 trials of 30 seconds each. For k=90 we have 2 recording sessions x ({greater than or equal to}2 sweeps) x 7 animals {greater than or equal to} 28 trials of 30 seconds each. Few recordings were removed due to artifacts. The actual numbers of trials used for every analysis is reported in the main text.

The Materials and Methods -> Experimental design -> Visual stimuli paragraph now reads:

Extracellular signals were recorded in response to abrupt reversals (1 Hz) of vertical square-wave gratings (spatial frequency, 0.06 c/deg; contrast levels adopted: [0,10,20,30,50,90]% ).

It was hard to find the source of the total number of recordings (e.g. Figure 2 C, left, 350 recordings across 12 animals). Our assumption was that this number would reflect 4 recording sessions per animal x 6 contrast levels (5 for 5 of the animals) x 12 animals, but that yields 268 recordings.

We thank the reviewers for pointing this issue out. The reviewer correctly pointed out the number of recording should have been 268 if all the animals would have undergone exactly 2 sweeps of contrasts each. In our experiments, instead, we assured to acquire at least two sweeps for each animal. In other words, some of the animals underwent an additional contrast sweep. The number of recordings reported in the text, furthermore, takes also into account the recordings discarded due to artifacts. 8 Fig 1b shows the scale in dB but dB normalization was not described in the methods.

We thank the reviewer for spotting this oversight. We added a sentence within the Materials and Methods -> Neurophysiological data analysis -> Spectral analysis paragraph:

The power spectral densities were converted in decibels (dB): *P**S**D**d**B* = 10*l**o**g*10(*P**S**D*).

Extracting broadband and narrowband gamma. Authors may want to consider modeling oscillatory and broadband gamma activities through an approach such as fooof (Donoghue et al., 2020, Nature Neuroscience); the current band limited approach matches previous studies but may lose some information.

We thank the reviewer for this observation. We think that approaches as foof are indeed highly valuable, and we have used them in previous works where the relevant frequency bands where not known a priori.

However, in the present work the comparison with the frequency bands already used in the experimental works we considered as references is the priority, hence we will keep the band limited approach currently used.

Fig 1B “narrow band (middle, black) and broad band (black). “ Should this be broad band (gray)?

We fixed the typo.

Code availability

Neither reviewer was able to access the code. It is necessary for code to be available, and we frequently

find that other researchers have difficulty implementing code. Please upload to an anonymized hub (e.g.

github) so the code can be tested. Alternatively you could remove the double blind.

See response to the first comment.

Reply letter references

Bastos AM, Briggs F, Alitto HJ, Mangun GR, Usrey WM (2014) Simultaneous Recordings from the Primary

Visual Cortex and Lateral Geniculate Nucleus Reveal Rhythmic Interactions and a Cortical Source for

Gamma-Band Oscillations. Journal of Neuroscience 34:7639-7644.

Battaglia D, Hansel D (2011) Synchronous Chaos and Broad Band Gamma Rhythm in a Minimal Multi-Layer

Model of Primary Visual Cortex Sporns O, ed. PLoS Comput Biol 7:e1002176.

Brunel N, Hakim V (1999) Fast Global Oscillations in Networks of Integrate-and-Fire Neurons with Low Firing

Rates. Neural Computation 11:1621-1671.

Ho J, Tumkaya T, Aryal S, Choi H, Claridge-Chang A (2019) Moving beyond P values: data analysis with

estimation graphics. Nat Methods 16:565-566.

Saleem AB, Lien AD, Krumin M, Haider B, Rosón MR, Ayaz A, Reinhold K, Busse L, Carandini M, Harris KD

(2017) Subcortical Source and Modulation of the Narrowband Gamma Oscillation in Mouse Visual

Cortex. Neuron 93:315-322.9

Senzai Y, Fernandez-Ruiz A, Buzsáki G (2019) Layer-Specific Physiological Features and Interlaminar

Interactions in the Primary Visual Cortex of the Mouse. Neuron 101:500-513.e5.

Storchi R, Bedford RA, Martial FP, Allen AE, Wynne J, Montemurro MA, Petersen RS, Lucas RJ (2017)

Modulation of Fast Narrowband Oscillations in the Mouse Retina and dLGN According to

Background Light Intensity. Neuron 93:299-307.

Timme NM, Lapish C (2018) A Tutorial for Information Theory in Neuroscience. eNeuro 5:ENEURO.0052-18.2018.

Victor JD (2002) Binless strategies for estimation of information from neural data. Phys Rev E 66:051903.

Welle CG, Contreras D (2016) Sensory-driven and spontaneous gamma oscillations engage distinct cortical circuitry. Journal of Neurophysiology 115:1821-1835.

Williams PL, Beer RD (2010) Nonnegative Decomposition of Multivariate Information. arXiv:10042515 [math-ph, physics:physics, q-bio] Available at: http://arxiv.org/abs/1004.2515 [Accessed May 28, 2021]
